# Recent Advances in Palladium-Catalyzed Isocyanide Insertions

**DOI:** 10.3390/molecules25214906

**Published:** 2020-10-23

**Authors:** Jurriën W. Collet, Thomas R. Roose, Bram Weijers, Bert U. W. Maes, Eelco Ruijter, Romano V. A. Orru

**Affiliations:** 1 Department of Chemistry and Pharmaceutical Sciences and Amsterdam Institute for Molecules, Medicines & Systems (AIMMS), Vrije Universiteit Amsterdam, De Boelelaan 1108, 1081 HZ Amsterdam, The Netherlands; j.w.collet@vu.nl (J.W.C.); t.r.roose@vu.nl (T.R.R.); b.weijers@vu.nl (B.W.); 2 Organic Synthesis, Department of Chemistry, University of Antwerp, Groenenborgerlaan 171, 2020 Antwerp, Belgium; 3 Organic Chemistry, Aachen-Maastricht Institute for Biobased Materials, Maastricht University, Urmonderlaan 22, 6167 RD Geleen, The Netherlands

**Keywords:** isocyanides, palladium, insertion reactions, heterocycles, catalysis

## Abstract

Isocyanides have long been known as versatile chemical reagents in organic synthesis. Their ambivalent nature also allows them to function as a CO-substitute in palladium-catalyzed cross couplings. Over the past decades, isocyanides have emerged as practical and versatile C_1_ building blocks, whose inherent *N*-substitution allows for the rapid incorporation of nitrogeneous fragments in a wide variety of products. Recent developments in palladium catalyzed isocyanide insertion reactions have significantly expanded the scope and applicability of these imidoylative cross-couplings. This review highlights the advances made in this field over the past eight years.

## 1. Introduction

### 1.1. Palladium Catalysis

Palladium-catalyzed cross-couplings were discovered close to 50 years ago. Since then, this class of transformations has evolved into one of the most widely applied reactions. Almost a quarter of all chemical reactions performed in medicinal chemistry are palladium-catalyzed cross-couplings [1]. The versatility of palladium-catalyzed couplings originates from the predictable and promiscuous catalytic reactivity of palladium. This also makes these catalysts ideal for cascade-type chemistry, where the active catalyst plays multiple roles [2]. Further developments in palladium catalysis have led to an ever-increasing scope of chemical methodologies, leading to milder conditions, lower catalyst loadings, and more robust and general cross-couplings. These aspects have made palladium-catalyzed cross-coupling reactions a critical staple in the development of chemical pathways to valuable fine chemicals.

### 1.2. Isocyanides

**Figure 1:**
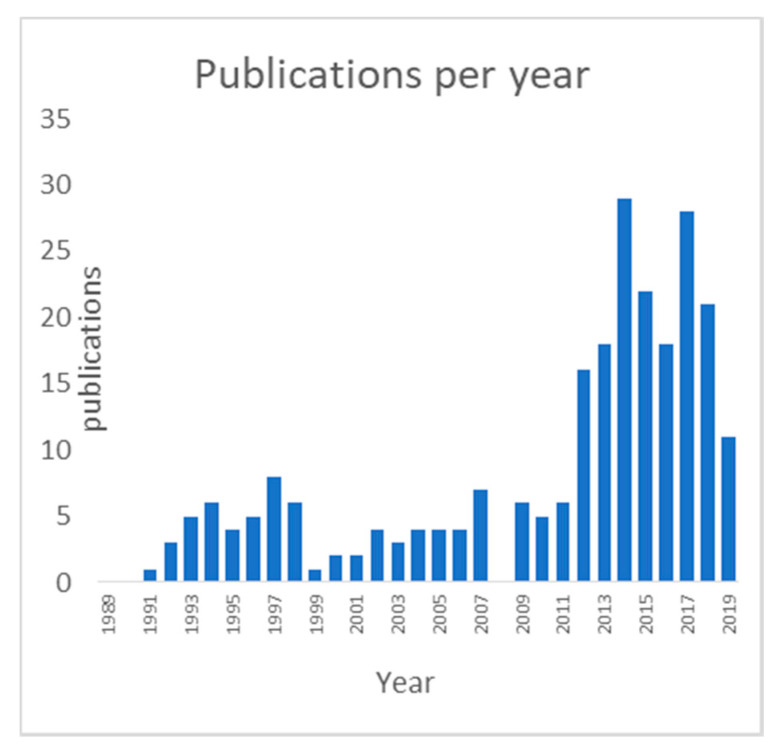
Publications per year reporting on reactions involving isocyanide reactant using palladium catalysis.

The usefulness of isocyanides in multicomponent chemistry (e.g., Passerini or Ugi-type reactions) is undisputed [3]. However, many other synthetic applications of these versatile building blocks have been known for a long time [4,5]. A relatively recent use of isocyanide building blocks combines their ambiphilic nature with well-defined transition metal-catalyzed cross-couplings. In these transformations isocyanides are inserted into a metal-carbon or metal-heteroatom bond [6,7,8], similar to better-known carbonylative cross-couplings [9,10]. The ability to insert an isocyanide into two coupling partners also makes this type of reactions interesting for the development of new types of multicomponent reactions (MCRs). While various metals are known to facilitate these ‘imidoylative’ cross-couplings, palladium is by far the most widely used metal in these transformations. In addition, the entire field of palladium-catalyzed isocyanide insertions has matured significantly over the past couple of years, with many new reactivities being reported (Figure 1). In this review we discuss all recent examples in which isocyanides are used as a reactant in palladium-catalyzed cross-couplings. We focus on cascade transformations or processes that couple three or more reactants in a single reaction.

This review augments our earlier comprehensive overviews of both palladium-catalyzed [7] and base metal-catalyzed [8] isocyanide insertions. As the synthetic applicability of these convergent synthetic strategies often hinges on the ability of the catalytic system to tolerate variations of the isocyanide substituent, we will especially discuss the isocyanide scope in each report. While tertiary isocyanides have traditionally been the main focus in most reports, this has changed more recently. Over the past decade, more robust catalytic conversions have been reported, often being much more tolerant of isocyanide input. Additionally, using palladium catalysis to insert an isocyanide, followed by the hydrolysis of the newly formed imine has rendered isocyanide insertions an effective tool for ’CO-free carbonylation,’ essentially utilizing the isocyanide as a carbonyl surrogate. Nickel-catalyzed polymerization of isocyanides to helical polyimines is extensively reported in literature [11,12]. Recently, significant advances have been made in using palladium catalysis for similar transformations to polyimines [13,14,15,16,17,18]. Due to the nature of this transformation, affording polymeric structures rather than small molecules, these examples will not be treated in this review.

## 2. Pd^0^-Catalyzed Isocyanide Insertions

### 2.1. Imidoylation Initiated by Oxidative Addition of Carbohalides

**Scheme 1:**
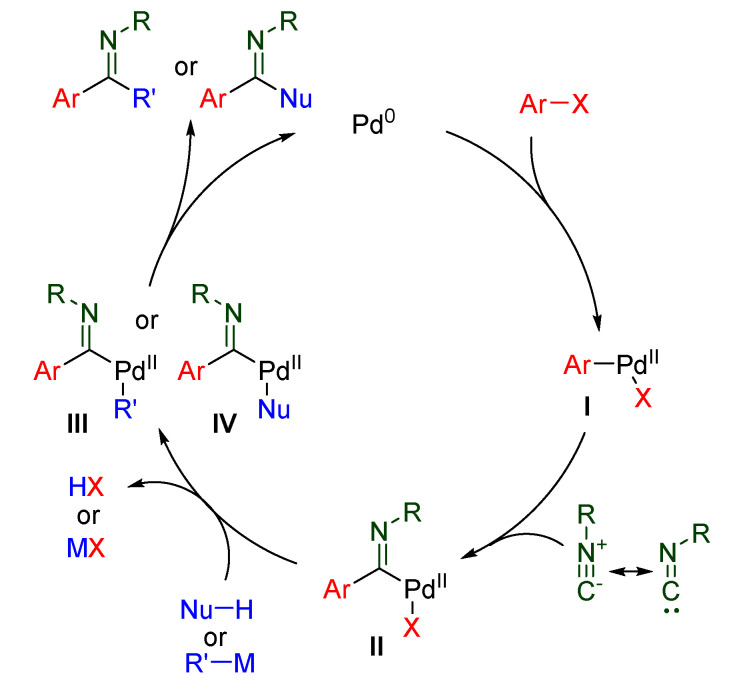
Typical catalytic cycle for redox-neutral Pd-catalyzed isocyanide insertion reactions.

Since the early discoveries in the 1970s, much of palladium catalysis still utilizes the propensity of aryl halides to undergo oxidative addition to an active Pd^0^ complex. This typically results in the formation of an aryl palladium complex **I** (Scheme 1). This complex can rapidly undergo 1,1-migratory insertion of isocyanides into the activated aryl palladium bond, furnishing imidoyl palladium complex **II**. Next, a ligand exchange can take place, either with ambient nucleophiles, or the complex **II** undergoes a transmetalation with metal-based coupling partners, resulting in the formation of complex **III** or **IV**. Subsequent reductive elimination then affords the imidoylative cross-coupling imine products, while regenerating the active Pd^0^ catalyst, thus closing the catalytic cycle. In the following sections, we discuss in more detail the catalytic processes, organized along the nature of the respective coupling partner.

#### 2.1.1. Cross-Couplings with Organometallic Carbon Nucleophiles

The ‘classic’ palladium-catalyzed cross-couplings almost all follow the typical pattern of oxidative addition, transmetalation, and reductive elimination, thus employing an external R’-M species as the coupling partner. Transmetalation is commonly an integrated step in the catalytic cycle of these processes, except perhaps for the well-known Mizoroki-Heck reaction, which is terminated by alkene insertion and β-hydride elimination. The first example of an imidoylation involving transmetalation by Mitiga et al. (imidoylative Stille coupling) [19] was followed by many studies that report major improvements in coupling partners, catalytic conditions, as well as tolerance for all coupling partners. Current methodologies have thus impressively matured over the past decade.

**Scheme 2:**
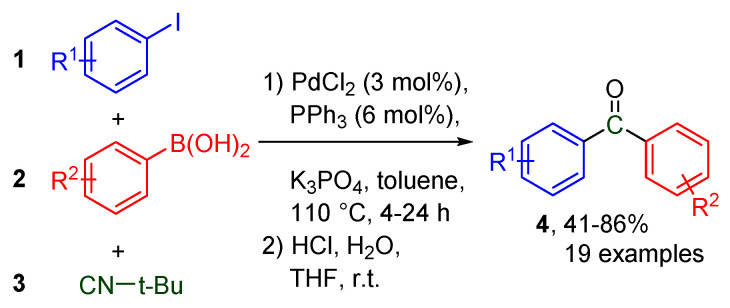
Biaryl ketone synthesis via imidoylative Suzuki-Miyaura reaction.

Although the extension of the well-known Suzuki-Miyaura reaction using isocyanides as starting material was already known [20], a new version of this cross-coupling reaction was introduced in 2014, describing an imidoylative synthesis of biarylketones **4** [21]. This redox-neutral cross coupling of aryl iodides **1**, aryl boronic acids **2** and *t*-BuNC (**3**) proceeds in good yields, although the use of sterically congested aryl iodides **1** leads to somewhat lower yields (Scheme 2). No attempts were made to investigate isocyanides other than *t*-butyl isocyanide **3**, as post-coupling acid hydrolysis cleaves of the isocyanide residue, liberating the ketone **4**.

**Scheme 3:**
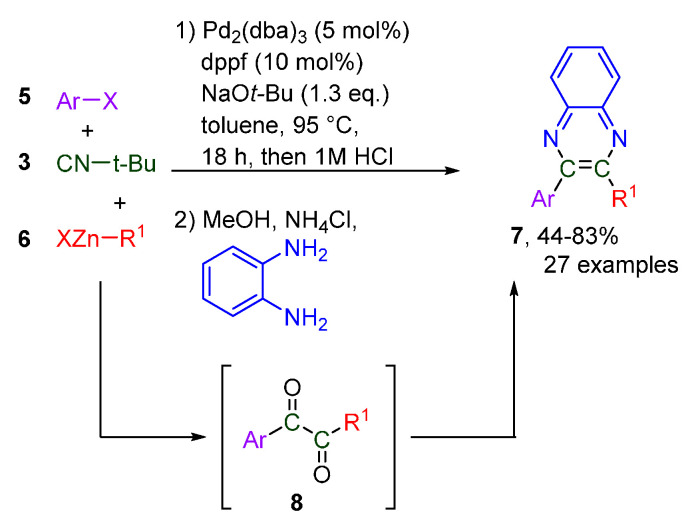
One-pot double imidoylative Negishi reaction and biscondensation with *o*-phenylenediamines to quinoxalines.

When the ‘classical’ palladium-catalyzed Negishi conditions are adapted by the addition of an isocyanide, a one-pot selective double isocyanide insertion was observed (Scheme 3) [22]. The choice of ligand is instrumental in the double insertion, as ligands other than dppf give mixtures of mono- and diketone products **8**. Although the catalytic system tolerates many different types of alkyl zinc reagents **6**, the use of isocyanides other than *tert*-butyl isocyanide resulted in poor to no conversion. This methodology was used to efficiently generate quinoxalines **7** via double condensation with *o*-phenylenediamine, in a three-step sequence, without intermediate purification (Scheme 3). 

**Scheme 4:**
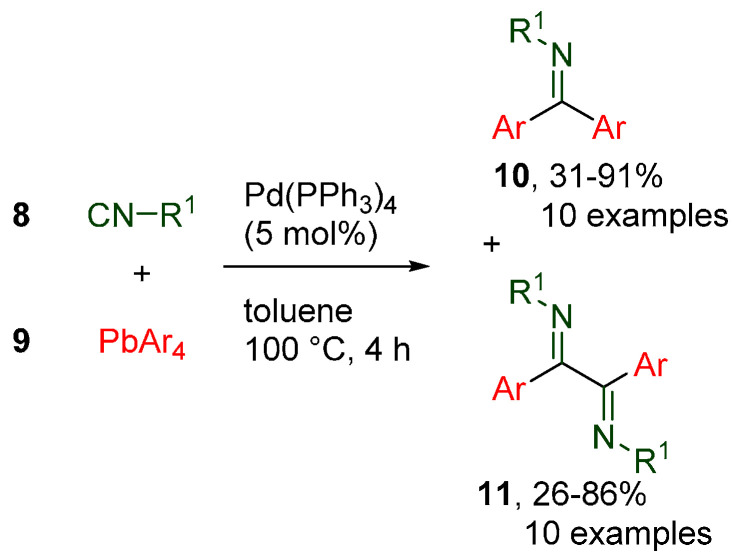
(Double) isocyanide insertion using tetraaryl lead reactants.

In an extension of this imidoylative cross-coupling using organozinc compounds (**6**) as coupling partners, the group of Ogawa reported a similar transformation with tetraaryl lead compounds **9** (Scheme 4) [23]. Generally, this reaction appears to be more difficult to control, typically affording a mixture of mono- and diimidoylated cross-coupling products **10** and **11**. However, this reaction proceeds without additives, and is compatible with aliphatic, benzylic, and aromatic isocyanides. Under the optimized reaction conditions, aliphatic isocyanides display a significant preference for coupling product diarylimines **10**. When electron-rich aromatic isocyanides were employed, this product distribution switched to diimidoylated structures **11** as the main product. Reactions with electron-deficient aryl isocyanides did not afford any isolable products.

**Scheme 5:**
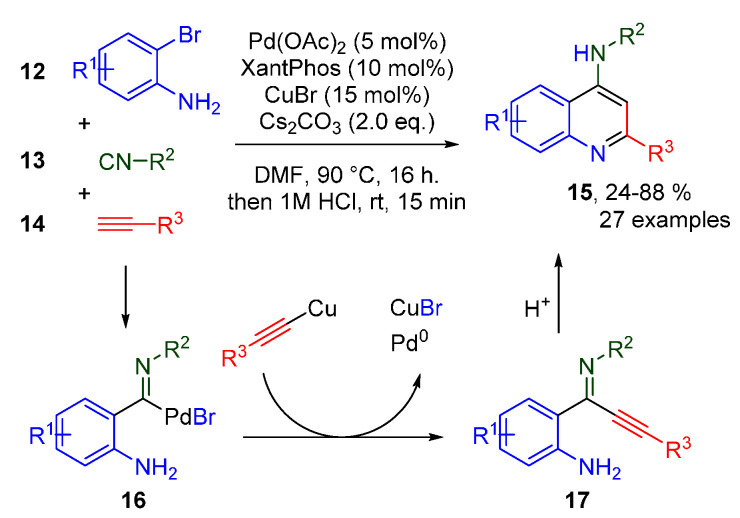
One-pot imidoylative Sonogashira reaction and acid catalyzed cyclization.

While an imidoylative Sonogashira reaction was already reported in 2013 [24], the initial publication only used *t*-BuNC as an isocyanide input, and hydrolyzed the formed ynimine in situ. In 2017, our group extended this methodology to include a nucleophile on the *o*-position of the aryl bromide substrate **12**, yielding 4-aminoquinolines (Scheme 5). Formation of the imidoylative Sonogashira intermediate **17** proceeds through transmetalation of an in situ formed copper acetylide to imidoyl palladium **16**. The afforded ynimine **17** readily undergoes acid-mediated intramolecular conjugate addition to directly afford medicinally valuable 4-aminoquinolines **15** [25]. The methodology is compatible with a range of different isocyanides, with secondary and primary isocyanides being well tolerated. Even secondary isocyanides, bearing an additional amine or carbamate moiety, were smoothly incorporated in this Sonogashira-type cross-coupling. The yields are inversely correlated to the electron density of the arenes, as decoration of either the 2-bromoaniline **12** or phenylacetylene **14** with electron-withdrawing groups resulted in lower yields, and more side reactions, most likely due to less facile, and more promiscuous cyclizations. A similar catalytic synthesis of thiochromen-4-ones was reported in 2018. [26]

#### 2.1.2. Cross-Coupling with Other Carbon Nucleophiles

Similar to the imidoylative C-C bond formations via transmetalation with an organometallic reactant, the past decade has seen a significant surge in the use of other carbon nucleophiles as coupling partners, typically formed in situ via deprotonation with readily available bases. The drawback is that this is restricted to active methylenes; additionally, the carbon coupling partner formed should be nucleophilic enough to interact with electrophilic palladium complexes. However, many advancements have been made recently, greatly enhancing the scope of such imidoylative cross-couplings. There are also other ways to generate carbon nucleophiles, involving electrophilic substitutions, e.g., from electron rich enamines or arenes, or via C-H activation.

**Scheme 6:**
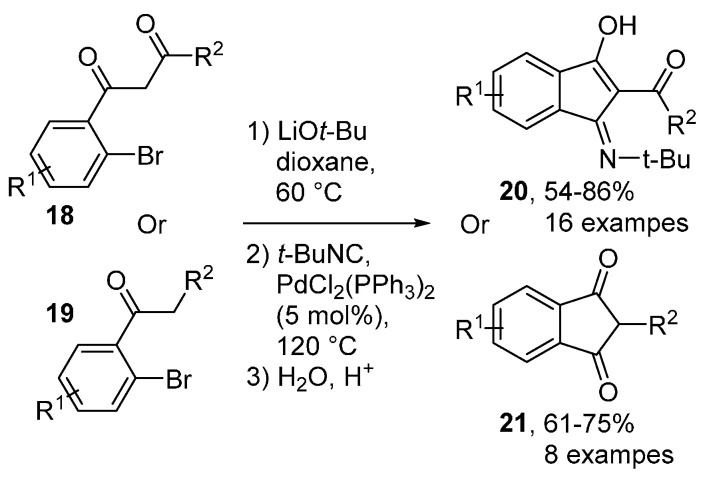
Utilizing enolates of 3-(2-bromoaryl)propanediones as coupling partners in imidoylative cyclizations.

In 2015, the group of Yang reported the Pd-catalyzed intramolecular C-C imidoylation with enolates of diones **18** to generate the 2-acyl-3-iminoindenes **20** [27]. This palladium-catalyzed isocyanide insertion is highly similar to those reported earlier by Ji et al., who reported an intramolecular isocyanide insertion and trapping of the imidoyl palladium intermediate with enols to afford the corresponding imidates [28]. Deprotonation by the stronger base *tert*-butoxide favors coupling with the enolate carbon over the enolate oxygen in moderate to good yields (Scheme 6). Similarly, omitting the distal ketone leads to a similar reactivity of *o*-bromoacetophenones **19**; however, all inputs were hydrolyzed with aqueous hydrochloric acid, leading to 1,3-indanediones **21** in acceptable yields (61–75%). 

**Scheme 7:**
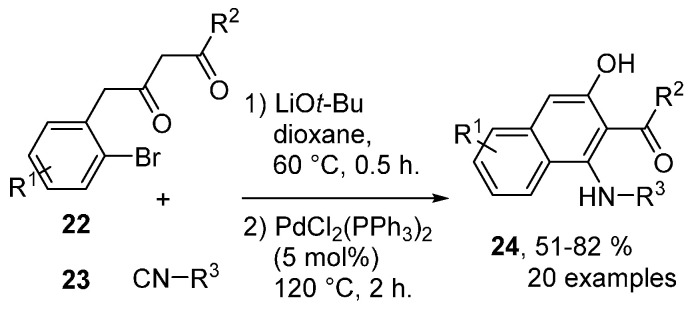
Utilizing enolates of 4-(2-bromoaryl)butanediones as coupling partners in imidoylative cyclizations.

This methodology was extended in the same year, introducing an additional methylene functionality to the substrate **22**. In this case, medicinally relevant amino-hydroxynaphthyl ketones **23** were obtained in moderate to good yields [29]. The use of tertiary isocyanides typically affords the 4-*tert*-butylaminonaphth-2-ols in good yields under the optimized conditions (Scheme 7). The authors did not fully investigate the scope regarding the isocyanide input, although a single cyclohexyl isocyanide entry afforded the corresponding naphthol **23** in a yield of 51%.

**Scheme 8:**
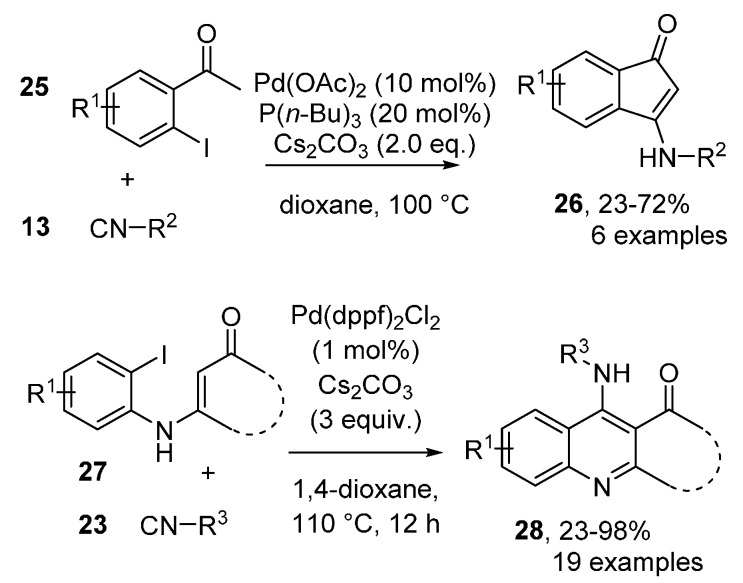
Utilizing enaminate of *N*-(2-iodoaryl)enaminones as coupling partners in imidoylative cyclizations.

The tether to the internal enolate can also be shortened, and still afford analogous products. In this manner, acetophenones **25** can also be coupled with the formed imidoyl palladium intermediate, affording aminoindenones **26** (Scheme 8). The scope of the reaction utilizing the corresponding enolate of acetophenones **25** is much less broad in terms of isocyanide substituents, as the yields of cyclic enaminones **26** drop significantly even when cyclohexyl isocyanide is used.

Another early example of imidoylation with a carbon coupling partner is shown in Scheme 8. The generated imidoyl palladium species is attacked by the electron-rich enamine carbon of enaminones **27**, which affords the medicinally interesting 4-aminoquinazoline derivatives **28** after reductive elimination [30]. The imidoylative cross-coupling proceeds with low catalyst loading, and generally affords annulated 4-aminoquinoline products in yields of around 90%, though the cyclization efficiency is greatly diminished if acyclic enaminones are used. The use of secondary isocyanides also reduces the yields, and no isolable quantities are formed when aromatic or benzylic isocyanides are employed.

**Scheme 9:**
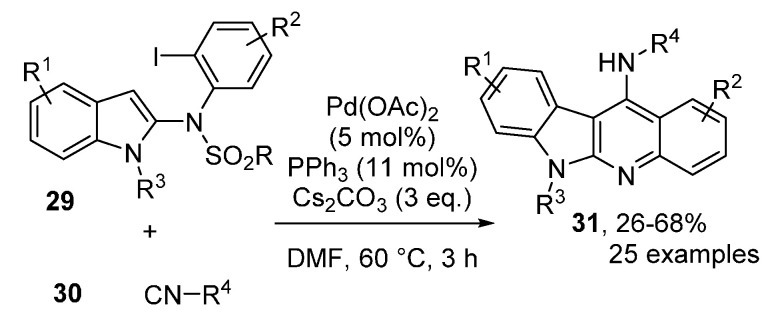
Utilizing an electron rich arene of 2-(2-iodoarylamino)indoles in imidoylative cyclizations.

Another example of the redox-neutral cross-coupling with carbon nucleophiles involves the synthesis of indoloquinolines **31** from 2-(2-iodoarylamino)indoles **29** and isocyanides **30** [31]. This reaction is postulated to proceed via isocyanide insertion into the generated aryl palladium bond, and subsequent electrophilic aromatic substitution at the highly electron-rich indole C3-position (Scheme 9). *N*-substituted indoloquinolines **31** were isolated successfully from tertiary and secondary aliphatic isocyanides. A single example was reported starting from 4-nitrophenyl isocyanide, although the corresponding indoloquinoline was formed in much lower yield (26%). The same group later facilitated the same reaction, with double isocyanide insertion, affording amide analogs of **31**, which show significant selective cytotoxicity for several cancer cell lines [32].

**Scheme 10:**
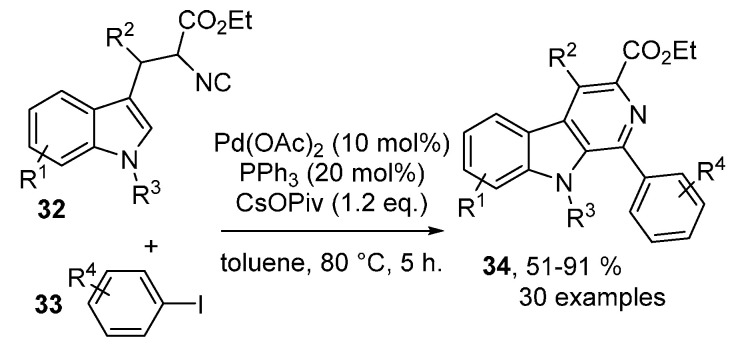
Intramolecular imidoylation of aryl iodides with isocyanotryptophanes.

An imidoylative cross-couplings using an isocyanide with a tethered carbon nucleophile is highlighted in Scheme 10. Using the readily available tryptophan-derived isocyanides **32**, a redox-neutral carboimidoylation was readily facilitated [33]. Regardless of the substituent pattern, all aryl iodides **33** afford β-carbolines **34** in good to excellent yields. The authors suspect that the initially formed dihydrocarbolines are oxidized under aerobic conditions during work-up to the medicinally valuable carbolines **34**.

**Scheme 11:**
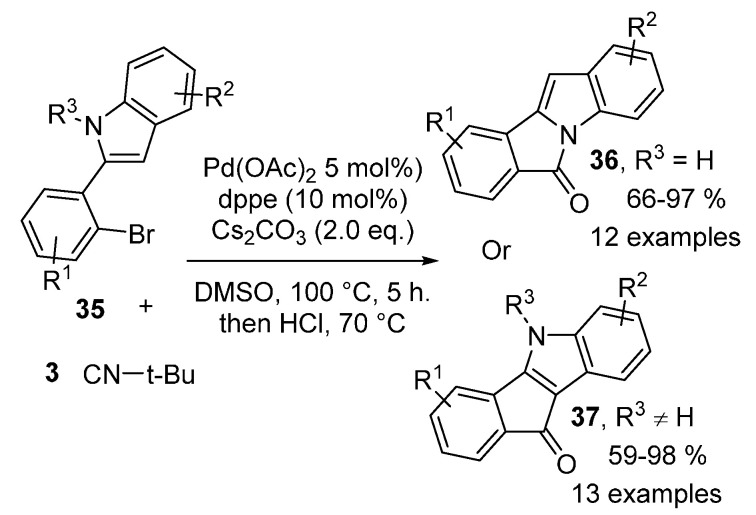
Substrate-dependent intramolecular carboimidoylation or aminoimidoylation.

A final example of using indoles as cross-coupling partners is shown in Scheme 11, in which the divergent reactivity of 2-(2-bromoaryl)indoles **35** was highlighted [34]. In the case of *N*-unsubstituted indole, all employed conditions favor the Buchwald-Hartwig-type amidination with the indole nitrogen, affording indoloindolones **36** by post-coupling hydrolysis of the amidine. Implementation of a *N*-substituent favors formation of indoloindenones **37** via a carboimidoylation rather than an aminoimidolyation, in comparable yields. In all cases, only *tert*-butyl isocyanide was used, and the product ketimines were hydrolyzed by addition of aqueous HCl.

**Scheme 12:**
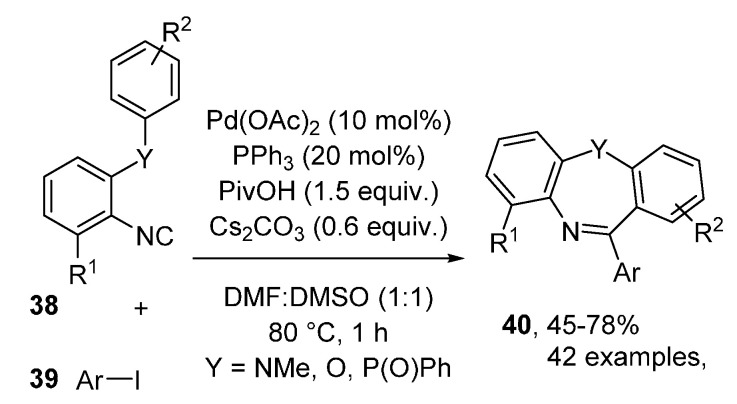
Utilizing an electron rich arene of 2-(aryloxy, arylamino or arylphosphino)aryl isocyanides in imidoylative cyclizations.

Recently, the group of Zhu extended this reactivity, reporting a direct imidoylative annulation of substituted aromatic isocyanides **38** and aryl iodides **39**, generating medicinally valuable dibenzazepines and dibenzoxazepines **40** (Scheme 12) [35]. The cross-coupling of *o*-isocyanodiphenylether affords significant amounts of double isocyanide insertion product. However, if 2,6-disubstituted phenyl isocyanides are used, formation of this side product is avoided, forming products **40** with surprising efficacy for a redox-neutral palladium-catalyzed aromatic isocyanide insertion. Dibenzodiazepines **40** (Y = NMe) were synthesized in similar yields via the same method. 

**Scheme 13:**
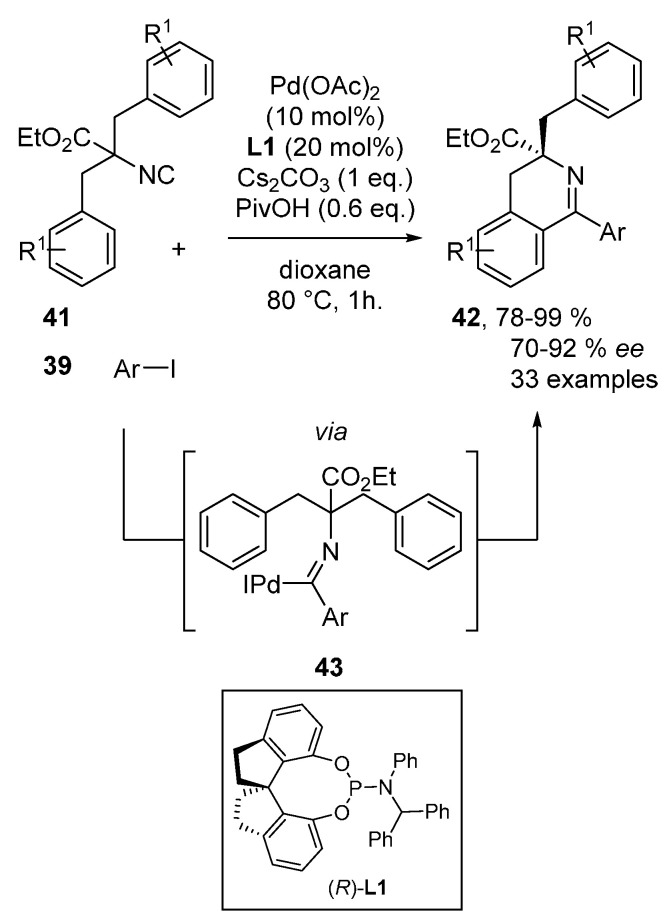
Enantioselective desymmetrization of dibenzyl isocyanoacetates in a Pd-catalyzed imidoylative annulation.

In 2017, the same group published an enantioselective desymmetrization of dibenzyl isocyanoacetates **41** [36]. Isocyanide insertion into the formed arylpalladium affords the intermediate imidoyl palladium species **43**, which undergoes an enantioselective carbocyclization to access the medicinally valuable dihydroisoquinolines **42** (Scheme 13). Utilization of the chiral spinol-derived ligand **L1** affords the products **42** in high yields, and typically in moderate to high *ee*’s. Additionally, a wide variety of aromatic substituents are tolerated on the dibenzyl isocyanoacetates **41**. 

The radical cyclization of *o*-isocyano biphenyls and related structures towards substituted phenanthridines was extensively reviewed by Studer in 2015 [37]. However, several palladium-catalyzed ionic syntheses of phenanthridines were also reported recently. In most of these cases, the mechanistic implications of the cycloaromatization step are not fully elucidated. These formal C-H functionalizations can either rely on a classic two step S_E_Ar, as several examples above, or have a more concerted character, bordering on electrocyclizations. 

**Scheme 14:**
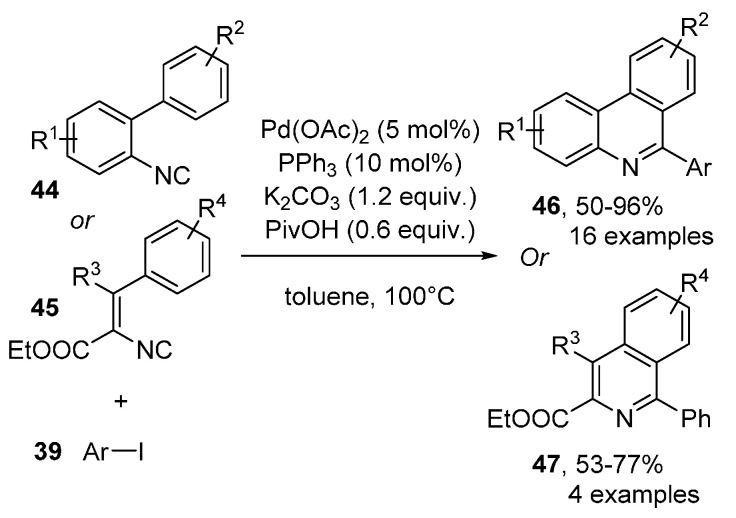
Intramolecular imidoylation of aryl iodides with biaryllisocyanide and (benzylidene)isocyanoacetates.

**Scheme 15:**
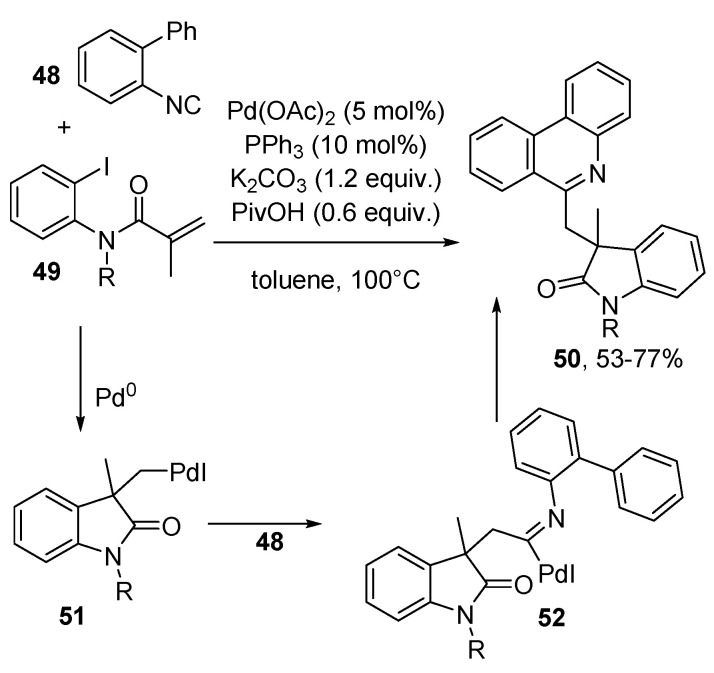
Intramolecular imidoylation of aryl iodides with biaryl isocyanide involving additional alkene insertion.

The redox neutral imidoylation of aryl iodides **39** with biaryl isocyanides **44** was reported in 2015 as a direct method of generating phenanthridines **46** in good to near-quantitative yields (Scheme 14). The reaction proceeds via isocyanide insertion into the formed arylpalladium species. Then, C-H functionalization affords the re-aromatized phenanthridines **48** [38]. Kinetic isotope effect experiments suggest that the annulation proceeds via C-H activation and reductive elimination, although an electrophilic aromatic substitution cannot be excluded. Changing the biaryl isocyanide to (benzylidene)isocyanoacetates **45** affords the corresponding isoquinolines **47** via a similar mechanism. Additionally, this method was also compatible with the alkylpalladate **51** generated by additional intramolecular proximal alkene insertion of methacrylamide **49**, affording chemically complex structures **50** in a one-pot fashion (Scheme 15). 

**Scheme 16:**
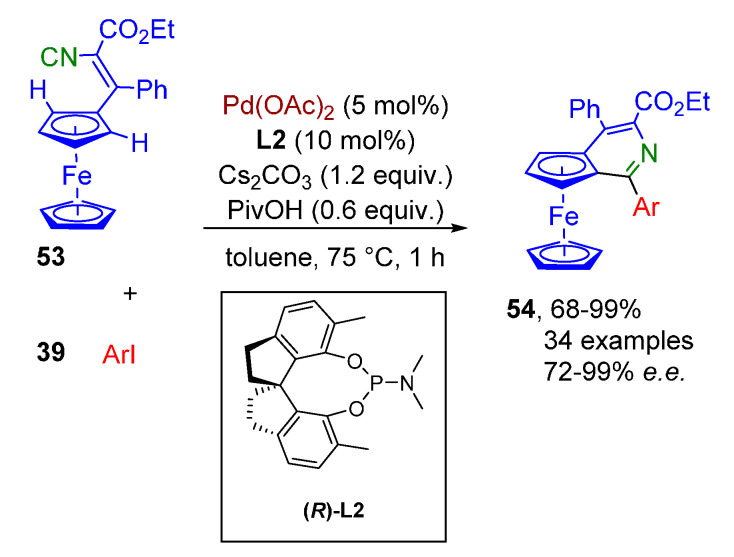
Asymmetric synthesis of pyrido-fused ferrocenes.

**Scheme 17:**
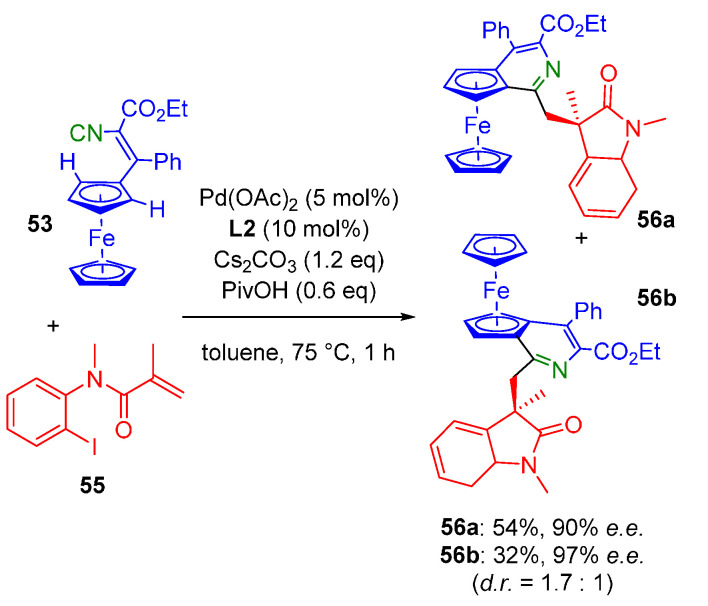
Asymmetric synthesis of indolinone substituted pyrido-fused ferrocenes.

The same group later reported an asymmetric variation of this transformation (Scheme 16). By using (ferrocenyl)vinyl isocyanides **53** and aryl iodides **39** in combination with chiral ligand **L2**, pyrido-fused ferrocenes **54** could be isolated in good to near-quantitative yields, with high *ee* [39] Similarly, with *N*-(2-iodophenyl)-*N*-methylmethacrylamide **55** additional alkene insertion occurs prior to isocyanide insertion. This cascade affords the highly complex structures **56** with high *ee* However, the chiral induction of the indolinone is moderate, leading to a *d.r.* of only 1.7:1 of **56a**:**56b** (Scheme 17).

#### 2.1.3. Cross-Couplings with Oxygen or Nitrogen Nucleophiles

In addition to transmetalation, the Buchwald-Hartwig reaction has received much attention by synthetic and medicinal chemists, as this reaction proved instrumental in the synthesis of many novel pharmaceutical scaffolds and their decoration [1]. The imidoylative variant of the Buchwald-Hartwig reaction involves nucleophilic attack on the transient imidoyl palladium species allowing for the formation of substituted imidates and amidines, and imines, depending on the nucleophile used. These functionalities are quite useful and ubiquitous in nature and pharmaceutical intermediates. Recent studies in the field of isocyanide insertions are directed towards further development of these imidoylative Buchwald-Hartwig type cross-couplings. To facilitate this, over the past couple of years there have been multiple reports on the mechanistic aspects of these reactions, allowing chemists to easier predict catalytic behavior and develop more effective catalysts [40,41,42]. 

**Scheme 18:**
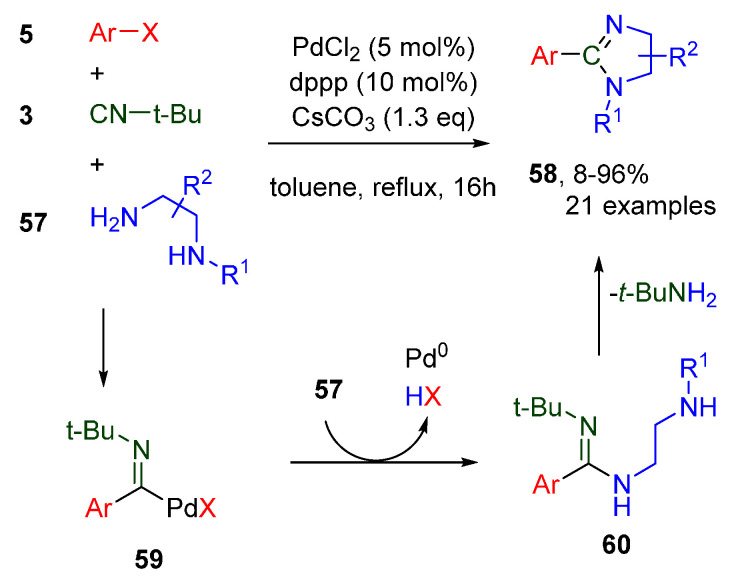
Cascade imidoylative Buchwald-Hartwig reaction and cyclization via transimination.

**Scheme 19:**
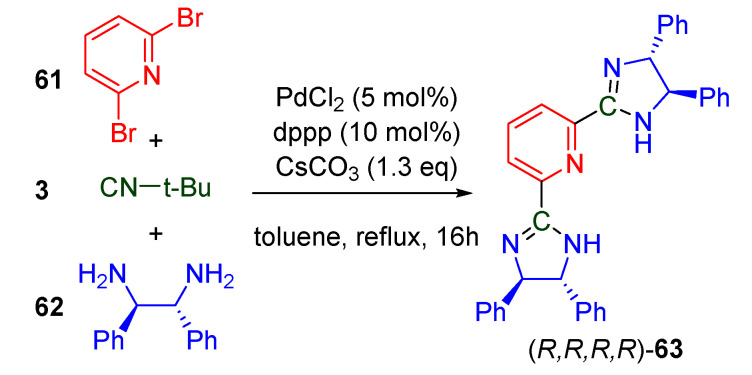
One-step bisimidoylative synthesis of chiral Pybim ligand.

In 2014, Schipman et al. extended the imidoylative Buchwald-Hartwig type cross-coupling to include the use of diamines **57** as coupling partners [43]. This reaction is indicative for imidoylative Buchwald-Hartwig couplings, and proceeds via the oxidative addition of an aryl halide **5** to in situ generated Pd^0^, after which a well-documented 1,1-migratory insertion of the isocyanide can take place. The transient imidoyl palladium intermediate **58** is readily attacked by ambient diamine **57**, which affords the amidine **60** after reductive elimination. This amidine smoothly undergoes an intramolecular transimination to afford 2-arylated imidazolines **58** (Scheme 18), releasing *t*-butylamine. The products are typically formed in good yields, although somewhat lower yields are reported for sterically hindered aryl halides, or less nucleophilic variations of diamine **57**, such as *o*-phenylenediamine. The authors also utilized this methodology to facilitate a direct synthesis of a complex chiral Pybim ligand **63** (Scheme 19).

**Scheme 20:**
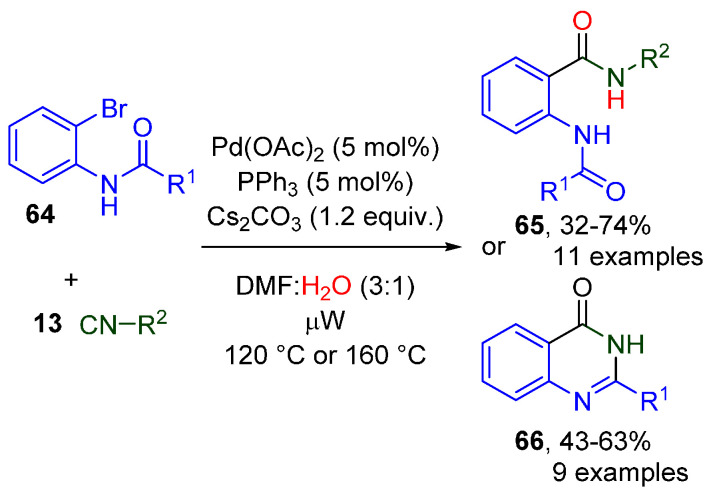
Temperature-dependent hydroxyimidoylation and subsequent condensation cascade.

A related method affords 2-substituted (3*H*)quinazolin-4-ones from *N*-acyl 2-bromoanilines **64**. [44] This method relies on the direct hydroxyimidoylation of the aryl halides with water as the nucleophile, affording bisamides **65** at 120 °C from either *tert*-butyl isocyanide or cyclohexyl isocyanide (Scheme 20). If the temperature is raised to 160 °C, the generated bisamide undergoes a cyclocondensation to quinolinones **66**, which is accompanied by spontaneous de-*tert*-butylation. It should be noted that similar one-pot hydroxyimidoylation/cyclization cascades are also reported in the syntheses of indolin-1-ones [45] and fused dihydropyrrolimines [46].

**Scheme 21:**
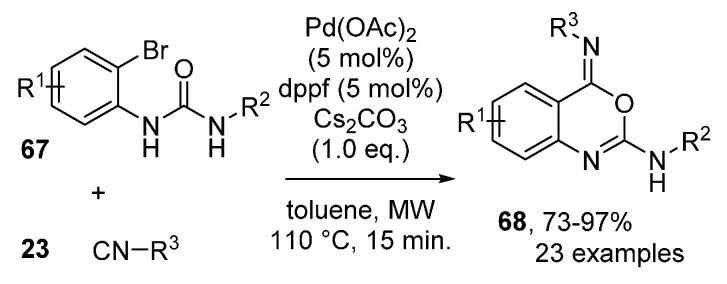
Intramolecular oxyimidoylation of (2-bromophenyl)ureas.

In a related process, the cyclooxyimidoylation of (2-bromoaryl)ureas **67** proceeds with full selectivity to the urea oxygen, which results in iminobenzoxazinones **68** (Scheme 21) [47]. In contrast to the couplings above, this transformation is highly compatible with various isocyanides, as aliphatic, α-acidic, and aromatic isocyanides all afford the iminobenzoxazinones **68** in high yields, regardless of the isocyanide substituent. The iminobenzoxazinones **68** are readily converted to the medicinally useful corresponding 2-aminobenzoxazinones via hydrolysis with aqueous HCl, or can be converted to the corresponding 2-aminoquinazolin-3*H*-ones through a piperidine-mediated Mazurkiewicz-Ganesan rearrangement, similar to earlier reports. [48] The same authors reported a similar reactivity for (2-bromophenyl)thioureas [49], and *N*-(2-bromoaryl)benzamides, yielding 2-aminothiazin-4imines 2-arylbenzoxazin-4-imines, respectively [50].

**Scheme 22:**
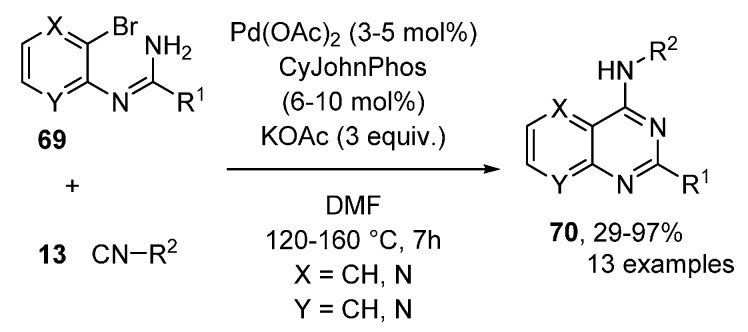
Fused quinazoline synthesis via intramolecular imidoylative Buchwald-Hartwig reaction of *N*-(pyridinyl)amidines.

Early in the development of this research field our group also reported an intramolecular imidoylative Buchwald-Hartwig reaction towards highly electron-deficient (aza)quinazoline **70** (Scheme 22) [51]. The transformation is successful in combination with secondary isocyanides and *tert*-butyl isocyanide, although no other tertiary isocyanides were investigated. The more electron-deficient *N*-(2-pyridyl)amidines **69** are more reactive towards imidoylative amidation than the regioisomeric *N*-(3-pyridyl)amidines, requiring lower temperatures, and affording the corresponding pyridopyrimidines **70** in higher yields. 

**Scheme 23:**
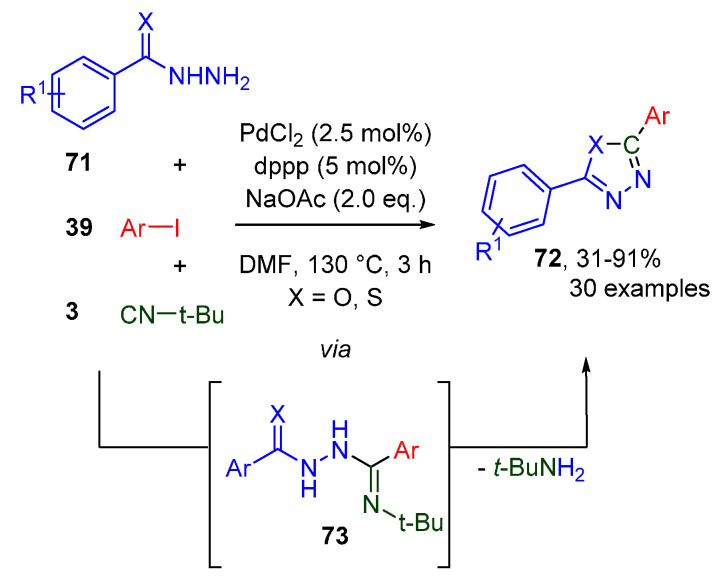
Cascade imidoylative Buchwald-Hartwig coupling with (thio)hydrazides followed by condensation (X = O, S).

The imidoylative Buchwald-Hartwig cross-coupling can also be employed in combination with (thio)hydrazides **71** in a redox-neutral synthesis of 1,3,4-oxadiazoles and thiadiazoles **72** [52]. As the isocyanide is only used as a C_1_ building block, the scope was left uninvestigated, and only *t*-BuNC was employed in this coupling (Scheme 23). The cross-coupling is also successful with aliphatic hydrazides, but results in a significantly reduced yield. The proposed intermediate (thio)acylamidrazone **73** undergoes a smooth cyclocondensation under the basic reaction conditions.

**Scheme 24:**
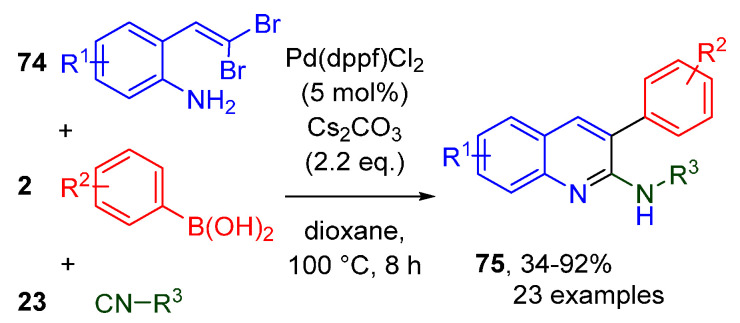
Sequential imidoylative Buchwald-Hartwig and Suzuki reaction on 2-amino-gem-dibromostyrenes.

Comparably, an initial Buchwald-Hartwig aminoimidoylation [53] can also be followed up by additional palladium-catalyzed cross-couplings in a one-pot fashion [54]. The multicomponent reaction (Scheme 24) generates 2-amino-3-bromoquinolines via an imidoylative Buchwald-Hartwig coupling, after which a subsequent Suzuki coupling takes place, affording 2-amino-3-arylquinolines **75**. Isocyanide insertion is fully selective; no indoles were formed through direct amination, and the follow-up Suzuki coupling proceeds completely without imidoylation. Although (*tert*-butylamino)quinolines **75** (R^3^ = *t*Bu) are typically isolated in yields over 70%, changing the isocyanide input significantly hampered the reaction. The use of cyclohexyl isocyanide led to an isolation of the corresponding product of a disappointing 34%, while 2,6-dimethylphenyl isocyanide gave only trace amounts of quinoline **75**. Similarly, in the absence of an internal nucleophile, hydroxyimidoylation of *gem*-dibromostyrenes to the corresponding alkynylamides is also possible [55].

**Scheme 25:**
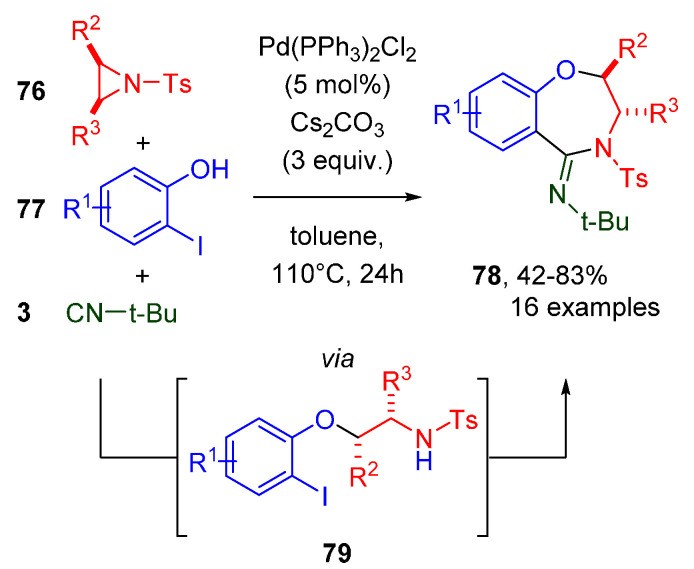
Imidoylative cross-coupling of 2-iodophenols and aziridines.

An interesting multicomponent cascade reaction with *N*-tosylaziridines **76** and *o*-iodophenols **77** affords access to benzoxazepines **78** [56]. The cascade is initiated by the base-promoted ring-opening of the aziridine by iodophenol **77** (Scheme 25). A subsequent intramolecular aminoimidoylation directly affords the polysubstituted benzoxazepines **78** in good yields, and in full *trans*-selectivity. The regioselectivity is controlled by sterics, opening the aziridine on the least hindered carbon. Again, reactions with tertiary isocyanides afford the corresponding benzoxazepines in good yields. The only secondary isocyanide (cyclohexyl isocyanide) that was tested afforded the product in a moderate 42%.

**Scheme 26:**
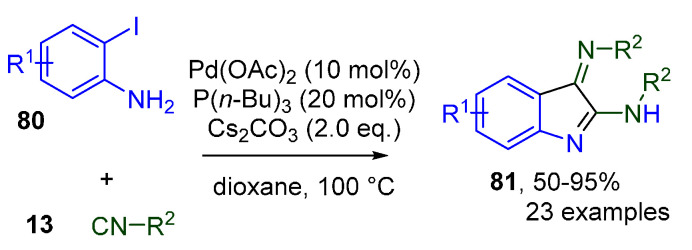
Aminoimidoylation of 2-iodoanilines with double isocyanide insertion.

An intramolecular Buchwald-Hartwig coupling with aryl iodides **80** bearing an internal nucleophile on the *ortho* position affords 2-amino-3-iminoindolenines **81** via double isocyanide insertion (Scheme 26) [57]. This reaction is fully compatible with tertiary, secondary, and aromatic isocyanides, but fails to afford any product **81** when primary aliphatic or α-acidic isocyanides are employed.

**Scheme 27:**
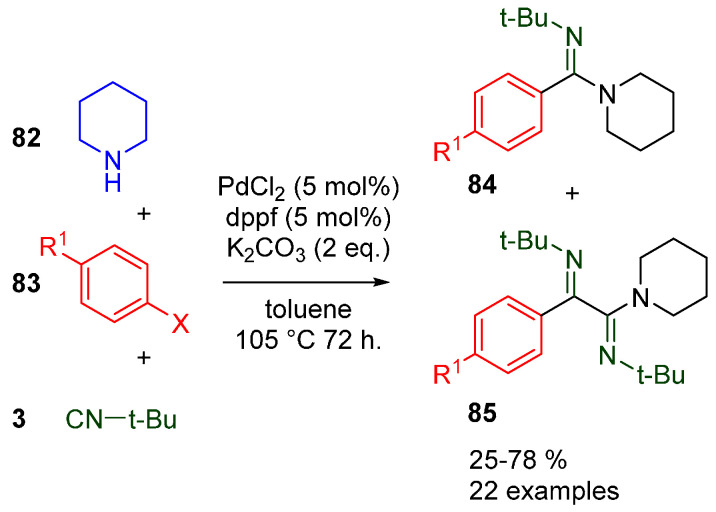
Single or double isocyanide insertion in Buchwald-Hartwig reaction.

In an intermolecular variant of this double insertion, the group of Kègl investigated the substituent effects of aryl halides **83** on the imidoylative cross-coupling with *tert*-butyl isocyanide **3** and secondary amines **82** [58]. Typically, this imidoylative Buchwald-Hartwig type multicomponent reaction affords the ketimine-amidine **85** in good yields, although in most cases a mixture of **85** and the amidine **84** is formed (Scheme 27). No linear Hammett correlation between the electron-donating or -withdrawing substituents and the yield or selectivity of this transformation was found. Interestingly, the authors note that the use of various bromoarene analogues of **83** (X = Br) typically favor a single isocyanide insertion towards benzamidines **84**, implying the halide plays a role in the mechanism. The broad substrate scope investigation was only performed with *tert*-butyl isocyanide and piperidine; other isocyanides showed no conversion under the reaction conditions.

**Scheme 28:**
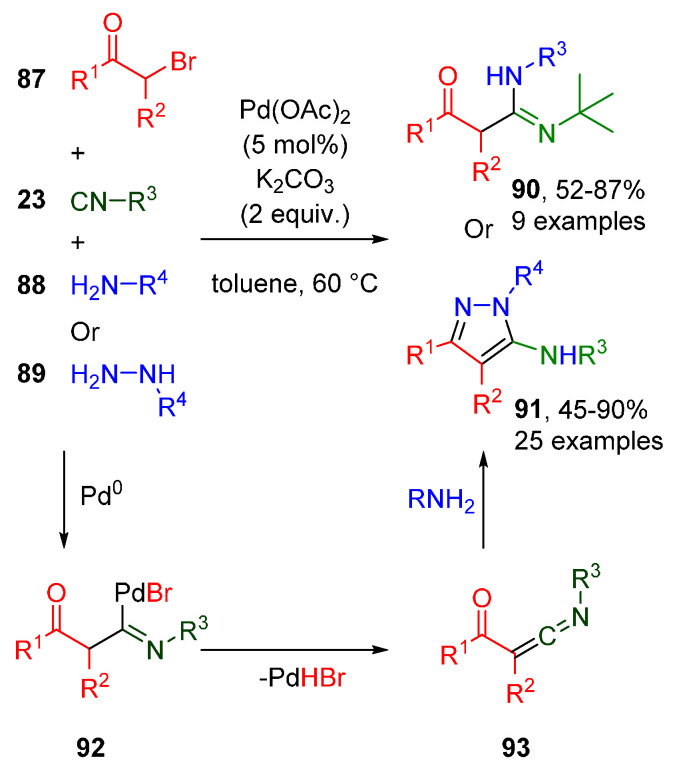
Pd-catalyzed ketenimine formation with alpha bromoketones and isocyanides and interception with nitrogen nucleophiles.

A Pd-catalyzed imidoylative MCR cascade to β-ketoamidines **90** or 5-aminopyrazoles **91** was reported by the group of Zhu [59]. Oxidative addition of the alkyl halide **87**, and subsequent isocyanide insertion is followed by β-hydride elimination of intermediate **92**, affording the ketenimine **93** as a common intermediate (Scheme 28). Subsequent addition of either amines **88** or hydrazines **89** as (bis)nucleophiles smoothly affords amidines **90** or 5-aminopyrazoles **91**, respectively. A single pyrazole **91** was synthesized using cyclohexyl isocyanide, although further investigations of the isocyanide scope were not performed. Additionally, the ketenimine can also be trapped by hydrazoic acid or Grignard reagents, highlighting the divergent nature of this transformation and the versatility of intermediate ketenimine **93**.

**Scheme 29:**
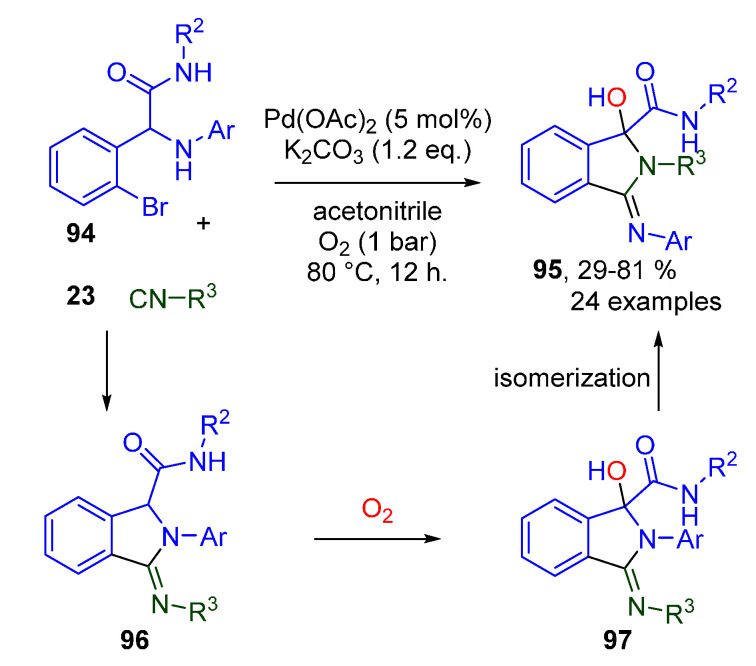
Imidoylative Buchwald-Hartwig reaction with Ugi products under O_2_ atmosphere.

The intramolecular imidoylative Buchwald-Hartwig reaction can also be performed under oxygen atmosphere yielding other products. Thus, imidoylation of the Ugi products **94** affords the isoindolimines **96**, which oxidize spontaneously under O_2_ atmosphere, to form α-hydroxy intermediate **97** (Scheme 29). Next, this intermediate isomerizes to form the thermodynamically more stable iminoisoindolinone derivatives **95** [60]. Curiously, the initial Buchwald-Hartwig imidoylation is successful under oxidative atmosphere, and does not require external stabilization by phosphine ligands. The transformation proceeds smoothly using aliphatic isocyanides, but is unsuccessful when aromatic isocyanides are employed, most likely due to their inherent instability.

**Scheme 30:**
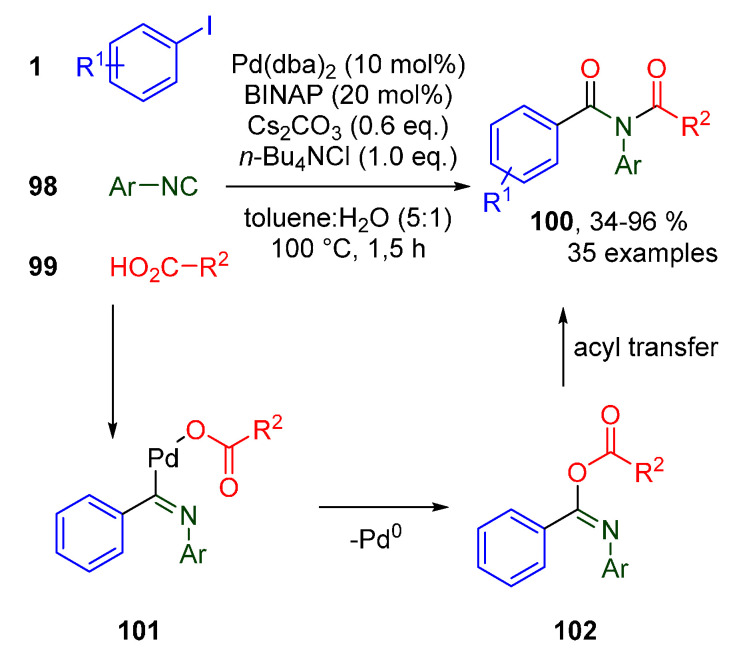
Imidoylative coupling of aryl iodides and carboxylic acids.

Carboxylic acids can also be used as nucleophiles, forming intermediate **101** (Scheme 30) [61]. Reductive elimination then affords the *O*-acylimidate **102** which undergoes a Mumm rearrangement, i.e., a spontaneous intramolecular acyl transfer into the *N*-arylimides **100**. Several examples were performed with bifunctional *o*-iodobenzoic acids, in which this imidoylative cross-coupling affords phthalimides in comparable yields to the untethered 3-CR variant. The reaction is highly compatible with aryl isocyanides, which is uncommon for redox-neutral palladium-catalyzed imidoylations. Additionally, through this method the authors facilitated late-stage diversification of well-known carboxylic acid pharmaceuticals (e.g., carboprofen, flurbiprofen).

**Scheme 31:**
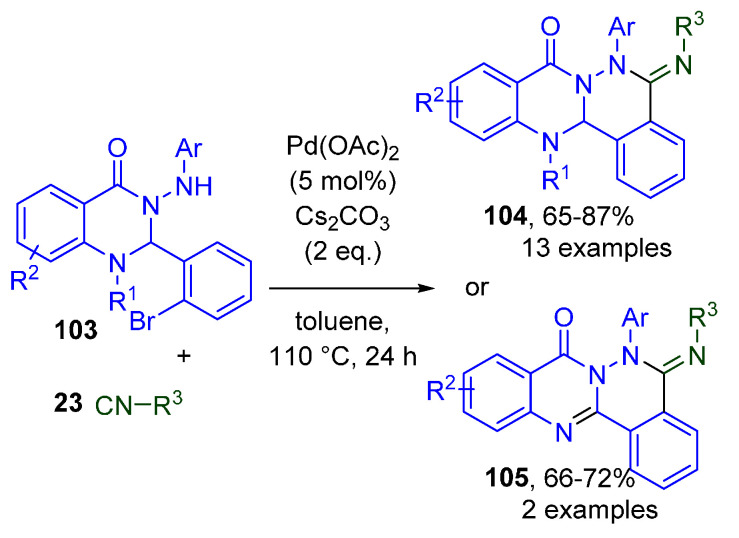
Imidoylative Buchwald-Hartwig cross-coupling of dihydroquinazolinones.

Further application of imidoylative Buchwald-Hartwig coupling includes the synthesis of fused tetracyclic scaffolds **104** (Scheme 31) [62]. The substrate dihydroquinazolinone **103** is formed in a cascade manner from isatoic anhydrides, benzaldehydes, and hydrazines. Fused hydrazide motifs have been reported for their analgesic and anti-inflammatory properties [63]. Although only *tert*-butyl isocyanide and cyclohexyl isocyanide were employed, both show similar reactivity in this redox-neutral imidoylation. Interestingly, when propargylated isatoic anhydrides are used to synthesize substrate **103** (R^1^ = CH_2_C≡CH), the product **104** undergoes a base-promoted, palladium-catalyzed cascade depropargylation, resulting in the aromatized quinazolin-4-ones **105** in good yields.

**Scheme 32:**
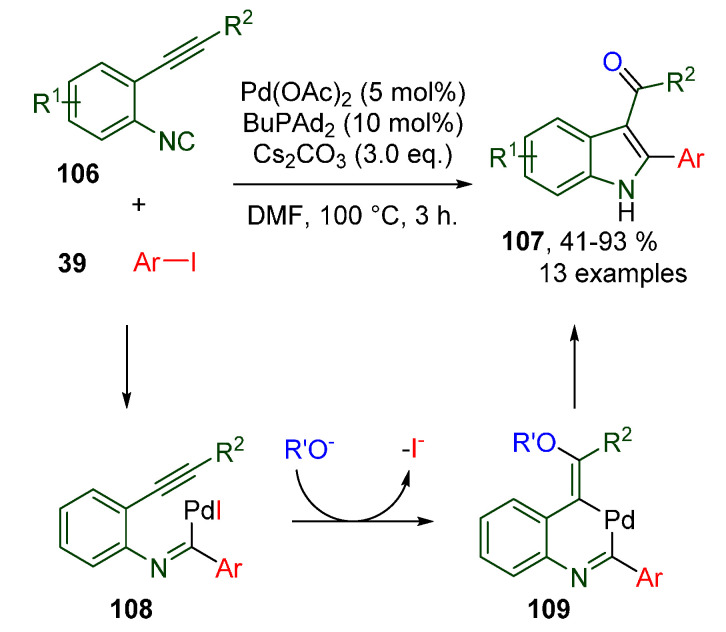
Isocyanophenylacetylene cyclization into 3-acylindoles. R’ = –H, –CO_2_^−^.

2-Alkynylated aromatic isocyanides **106** occasionally react in redox-neutral palladium-catalyzed isocyanide insertions (Scheme 32) [64]. The only two variations of aryl isocyanide substitutions that were investigated indicate the reaction is less efficient when electron density is withdrawn from the isocyano moiety. The isocyanide insertion of isocyanophenylacetylene **106** is followed by a 6-*exo*-dig cyclopalladation of the generated intermediate **108**. The authors also showed that oxygen is most likely introduced either by residual water, or the carbonate base, which also liberates 3-acylindoles **107** upon hydrolysis. 

**Scheme 33:**
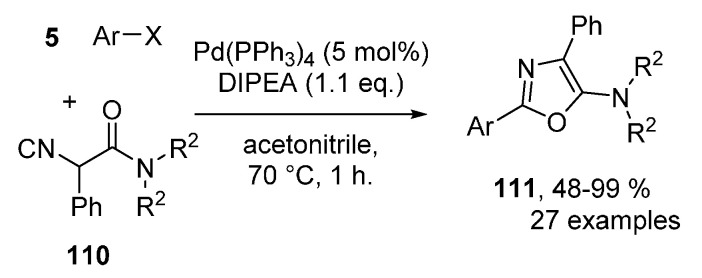
Imidoylative Buchwald-Hartwig cross-coupling with isocyanoacetamides.

Intramolecular trapping of formed imidoyl palladium species by a nucleophile-bearing isocyanide **110** affords the polysubstituted oxazoles **111** (Scheme 33) [65]. While the reaction is compatible with multiple aryl halides **5**, and typically affords the corresponding oxazoles in good yields, the isocyanoacetamide requires a tertiary amide. Further α-substitutions of the isocyanoacetamide were not investigated. Additionally, replacing the aryl halide with vinyl triflates or alkenyl bromides also generate structural analogues of oxazoles **111** in good yields via the same mechanism. An oxidative homocoupling of isocyanides **110** to afford bisoxazoles was reported later that year [66]. 

### 2.2. Imidoylation Initiated by Oxidative Addition of Oximes

Aside from the use of aryl halides as preoxidized substrates, recent reports have repeatedly included the propensity of oxidized amines and imines to undergo oxidative addition to a Pd^0^ catalyst. These acyloximes or acyloxyamines readily afford a *N*-Pd active site for isocyanide insertion, leading to more diverse, nitrogeneous cross-coupling products.

**Scheme 34:**
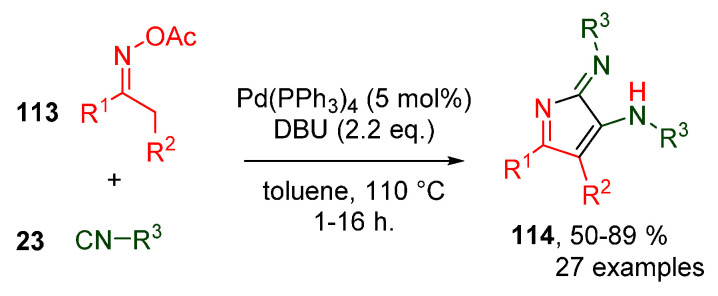
Intramolecular double imidoylation of *O*-acetyloximes.

The combination of *O-*acetyloximes **113** with isocyanides was first reported by Wang et al. in 2017. Utilizing the inherent nucleophilicity of enamines, the intramolecular double imidoylation of **113** affords the 3-aminopyrrol-2-imines **114** (Scheme 34) [67]. The method is tolerant towards aliphatic and aromatic isocyanides, although utilization of aromatic isocyanides tends to afford the products **114** in moderately lower yields, and typically requires extended reaction times. 

**Scheme 35:**
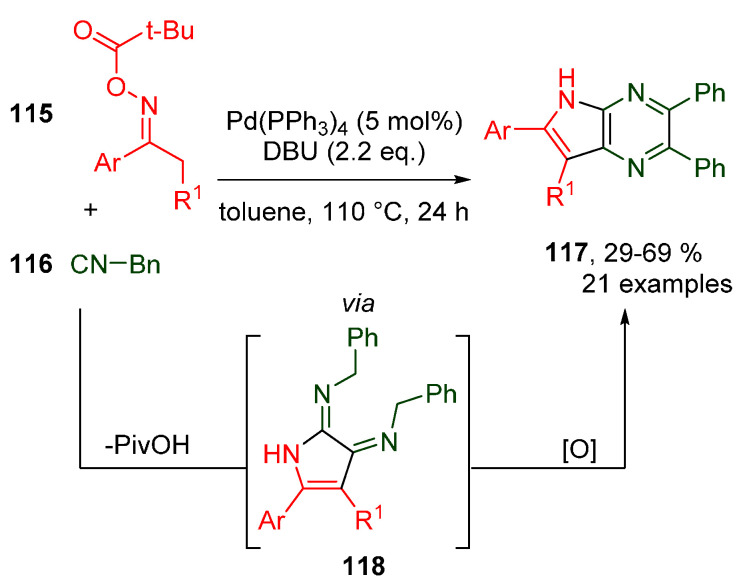
Imidoylation of *O*-acyloximes followed by dehydrogenative homocoupling.

The same group later exploited this reliable reactivity of isocyanide insertion into *O*-acyloximes to facilitate the curious dehydrogenative insertion of benzyl isocyanide **116** into these oximes **115** (Scheme 35). This affords the pyrrolediimines **118**, which undergo in situ dehydrogenative homocoupling, leading to the diphenylated pyrroloquinoxalines **117** in surprisingly good yields (29–69%) [68]. The benzylic isocyanide substituent is required, as the reaction fails with α-acidic isocyanides. The cross-dehydrogenative homocoupling of the benzylic substituents does not require an external oxidant.

**Scheme 36:**
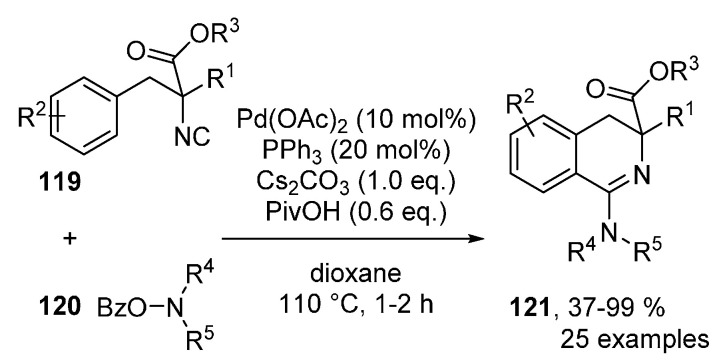
Imidoylative coupling of tertiary isocyanoacetates and *O*-benzoylhydroxylamines.

The methodology mentioned in Scheme 13 was later expanded by exploiting this oxidative addition of *O*-benzoyloxyamines **120** to the active Pd^0^ catalyst, and subsequent isocyanide insertion to generate the corresponding imidoyl palladium species (Scheme 36) [69]. Pivalate-assisted C-H functionalization of the tethered arene and reductive elimination affords the 1-aminodihydroisoquinolines **121**, typically in good yields. In this case, no asymmetric variations were attempted. The efficacy of this transformation is not hampered by the introduction of electron-withdrawing groups on the arene. No secondary isocyanoacetates (R^1^ = H) were reported. Additionally, benzoylhydroxylamines derived from primary amines were not tested under these conditions.

**Scheme 37:**
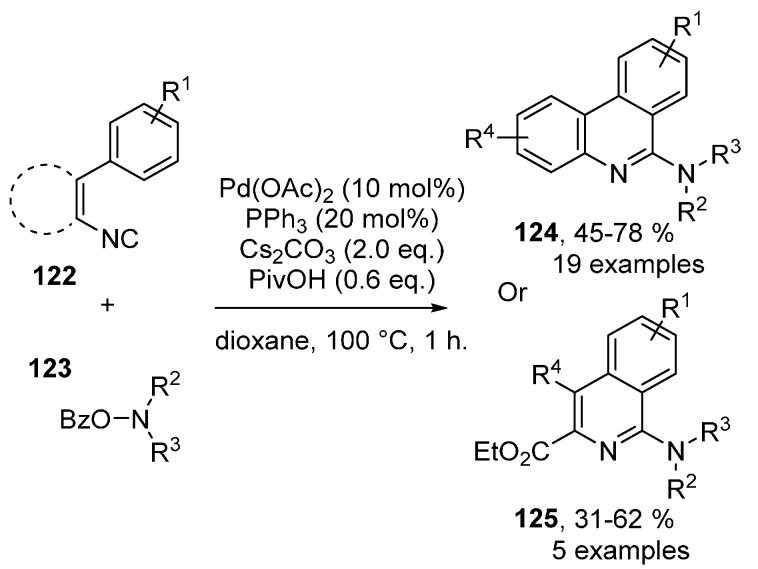
Intramolecular imidoylation of *N*,*N*-disubstituted *O*-benzoylhydroxylamines with biaryllisocyanide and (benzylidene)isocyanoacetates.

Related to the above-mentioned phenanthridines synthesis (Scheme 15), but initiated by the oxidative addition of *O*-benzoyloxyamines **123** to Pd^0^, 6-aminophenanthridines **124** or 1-aminoisoquinolines **125** are rapidly formed through a subsequent imidoylation and electrophilic aromatic substitution cascade [70]. Although the nature of the isocyanide substituents are of little consequence to the overall yield of these transformations, the reaction is only successful when *N*,*N*-disubstituted benzoyloxyamines are employed introducing tertiary amines on the cores (Scheme 37).

### 2.3. Imidoylation Initiated by Oxidative Addition of Allyl(pseudo)halides

**Scheme 38:**
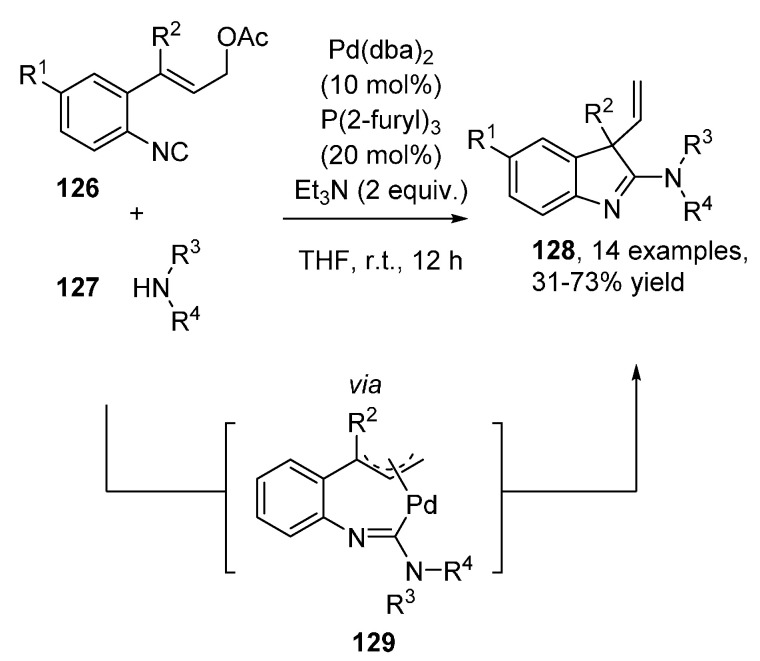
Intramolecular imidoylative Tsuji-Trost coupling with amines.

The first reported palladium-catalyzed imidoylation using allylic (pseudo)halides to initiate isocyanide insertion involves the synthesis of 3*H*-indolamines **128**, bearing a quaternary carbon center at C3, utilizing complex bifunctional 2-(3-acetyloxypropen-1-yl)aryl isocyanide **126** as a coupling partner (Scheme 38) [71]. The use of l-proline methyl ester as an amine component led to moderate stereochemical induction, affording **128** in 44% yield, as a 3:1 mixture of diastereomers.

**Scheme 39:**
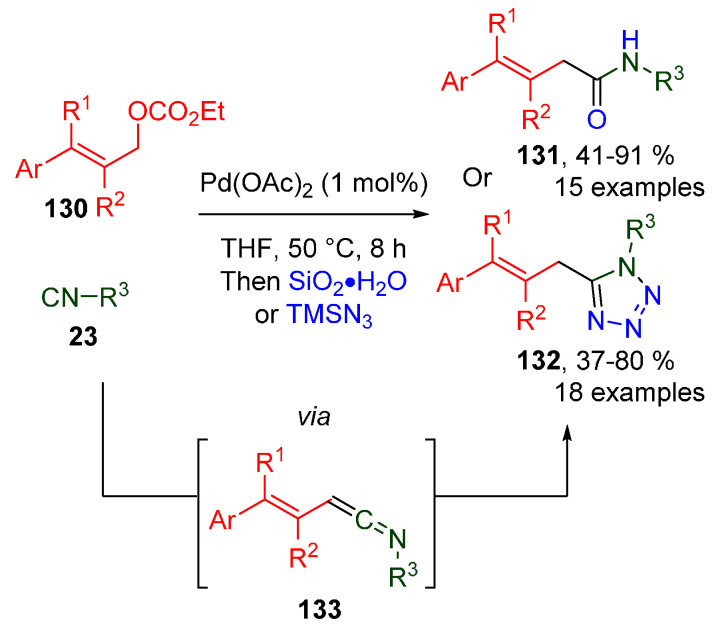
Homologation of allyl carbonates into allylic amides and 1H-tetrazoles via ketenimine formation.

The first intermolecular cross-coupling involving a π-allylpalladium complex and isocyanides was reported by Zhu et al. in 2016 [72]. They used allylic carbonates **130** to facilitate isocyanide insertion to give the corresponding vinylic ketenimines **133** (Scheme 39), which were subjected to hydration or formal [3 + 2] cycloaddition with azides in a one pot fashion to afford β,γ-unsaturated amides **131** or tetrazoles **132**, respectively. The reaction proceeds under remarkably mild conditions, and the products are typically isolated in good yields. As the product regiochemistry is under thermodynamic control, substitution at the R^1^ or R^2^ position can lead to formation of some regioisomers, or *E/Z*-diastereoisomers. While the authors report that the reaction is compatible with secondary and even aromatic isocyanides, these examples were not included in the initial publication.

**Scheme 40:**
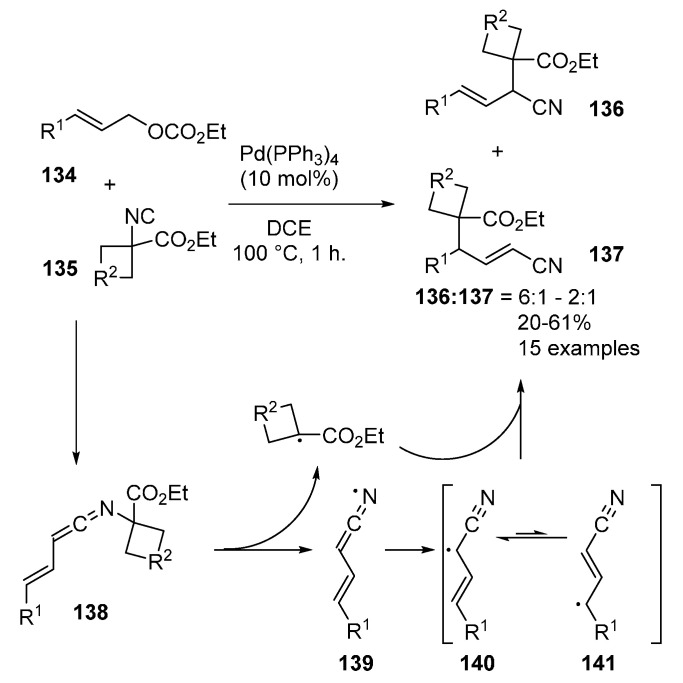
Tsuji-Trost imidoylation followed by radical rearrangement into cyanides.

An uncommon cyanation via isocyanide insertion into activated allylic (pseudo)halides can also proceed via a radical mechanism (Scheme 40). In a recent report by Zhu et al., the interesting conversion of allylic carbonates **134** and tertiary cyclic isocyanides **135**, bearing an α-ester moiety, was described [73]. The ketenimines **138** are formed, similar to Scheme 39, and are postulated to undergo homolysis to generate radical pair **140** and **141**. The rapid isomerization is most likely responsible for the mixture of isomers **136** and **137** formed. 

**Scheme 41:**
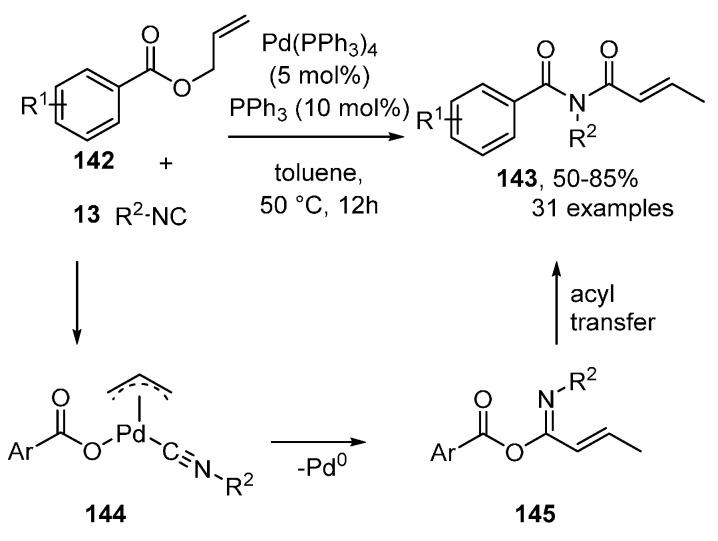
Imidoylative conversion of allyl benzoates into *N*-benzoyl-*N*-substituted crotonimides.

In 2017, Liang et al. reported a curious transformation, wherein readily available allyl benzoates **142** are readily converted to the corresponding *N*-benzoyl-*N*-substituted crotonimides **143** (Scheme 41) [74]. The reaction conditions are compatible with secondary and primary aliphatic isocyanides. Extensive mechanistic investigations were not performed, but the authors propose an isocyanide insertion into allylic Pd intermediate **144**, followed by reductive elimination to afford imidate intermediate **145**. This imidate undergoes an intramolecular acyl migration via Mumm rearrangement to afford the product imides **143**. The reductive elimination, generating conjugated intermediates such as **145**, has not been reported anywhere else, although similar reactions have been published over the past couple of years [71,72,73,75,76]. The authors make no mention of a possible hydride transfer. However, a post-coupling “chain-running” allylic isomerization of vinylacetimide towards the more thermodynamically stable conjugated crotonimide seems likely. These isomerization processes can also be catalyzed by palladium [77]. 

**Scheme 42:**
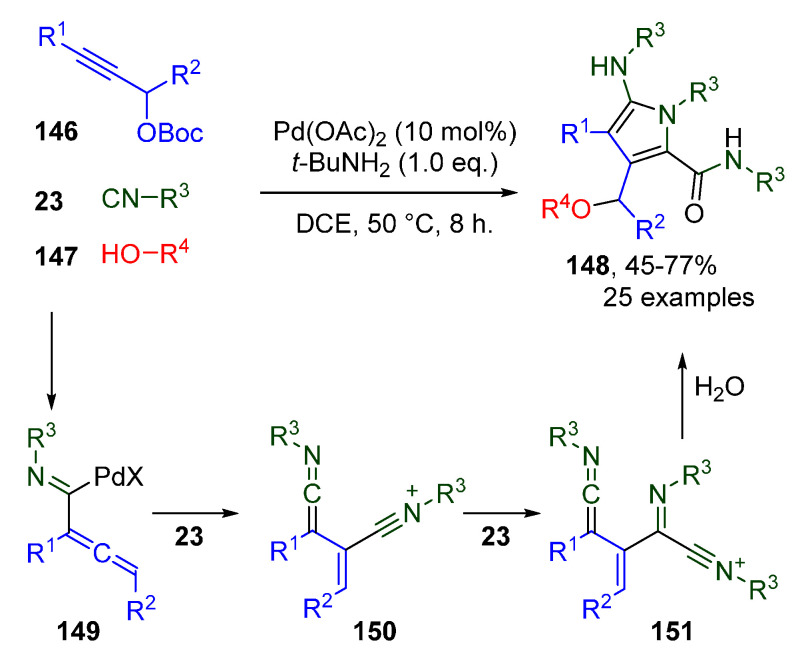
Aminopyrrole synthesis by triple isocyanide insertion cascade.

Zhu et al. reported one of the first controlled palladium-catalyzed triple isocyanide insertion reactions, facilitating the synthesis of highly decorated aminopyrroles **148** [78]. By extending the previously noted Tsuji-Trost activation of allylic acetates to propargylic carbonates **146**, an allenylpalladium species is formed that can undergo isocyanide insertion, providing intermediate **149** (Scheme 42). Attack of a second isocyanide **23** forms an electron-deficient nitrilium species **150** that is susceptible to external addition of yet another isocyanide, generating the postulated intermediate **151**. Only now is the sequence terminated by the addition of nucleophilic alcohol **147**, which performs a conjugate addition to form the aminopyrroles **148**. Only tertiary isocyanides were used in this triple insertion process.

### 2.4. Imidoylation Initiated by Oxidative Addition of Activated Sulfur Compounds

Imidoylative variants of less common palladium-catalyzed cross-coupling reactions are also reported. In this respect, the Liebeskind-Srogl coupling, typically involving an activated thioether or thioester, is an interesting example [79,80]. Although early reports rely on both palladium and copper catalysis, new versions of this reaction typically only require either copper or palladium catalysts. The scope of this cross-coupling has recently been extended to include isocyanides, although all imidoylative Liebeskind-Srogl-type reactions reported to date only insert an isocyanide into the activated substrate, retaining the leaving group, and therefore, they are formally not cross-coupling reactions. 

**Scheme 43:**
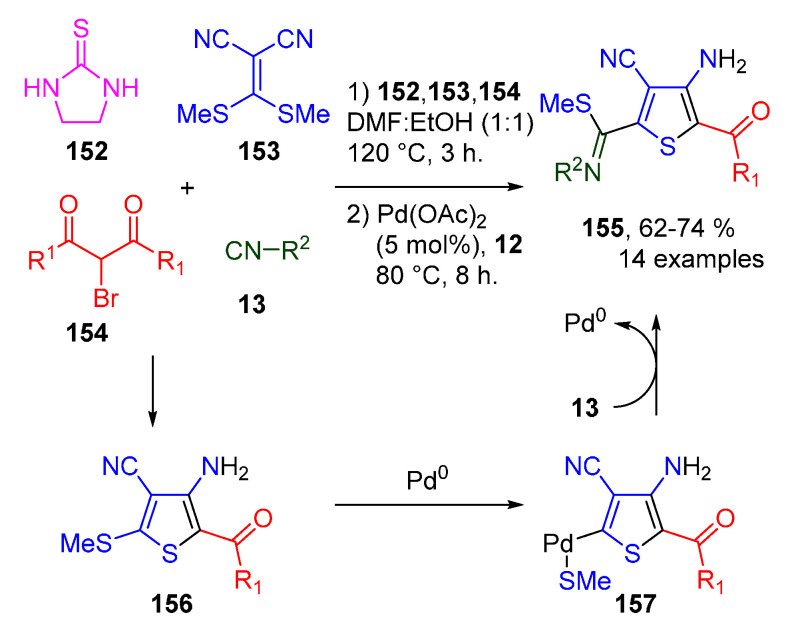
One-pot, two-stage imidoylative Liebeskind-Srogl coupling.

An early example (2017) of such an imidoylative Liebeskind-Srogl coupling was performed in a one-pot fashion as depicted in Scheme 43 [81]. The thiophene **156** is formed via an *S*-demethylation/Thorpe-Ziegler cyclization [82], and is directly subjected to the imidoylative reaction conditions. The reaction reliably affords the thioimidate **155** in 62–74% yield. Even when using cyclohexyl, or 2,6-dimethylphenyl isocyanide, the yields were similar to the reactions performed with the benchmark *tert*-butyl isocyanide. As this transformation is not affected by the free amine on the intermediate thiophene **156**, the product thioimidates could also be further derivatized by Cu/Pd-cocatalyzed amination of arylboronic acids, further increasing the chemical complexity in a one-pot, three-three stage reaction sequence.

**Scheme 44:**
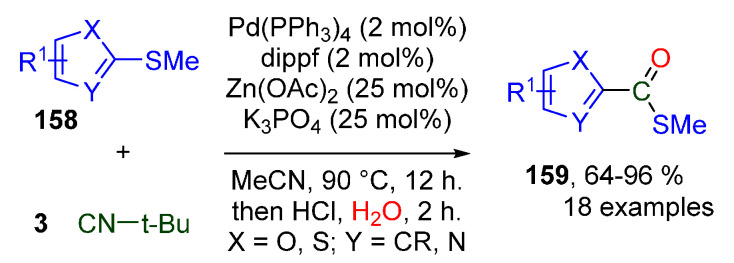
Imidoylative Liebeskind-Srogl coupling of methyl heteroaryl thioethers followed by in-situ hydrolysis of the thioimidate yielding thioesters.

Methyl heteroaryl thioethers **158** can be subjected to imidoylative conditions to selectively afford thioesters **159** (Scheme 44) [83]. It should be noted that without the Zn(OAc)_2_ additive no scrambling of various mixed methyl- and ethyl thioethers occurred, which suggests that the Lewis acidic zinc ion activates the thioether towards oxidative addition to the palladium catalyst. As the thioimidate product is hydrolyzed, only *tert*-butyl isocyanide was used in the reported examples.

**Scheme 45:**
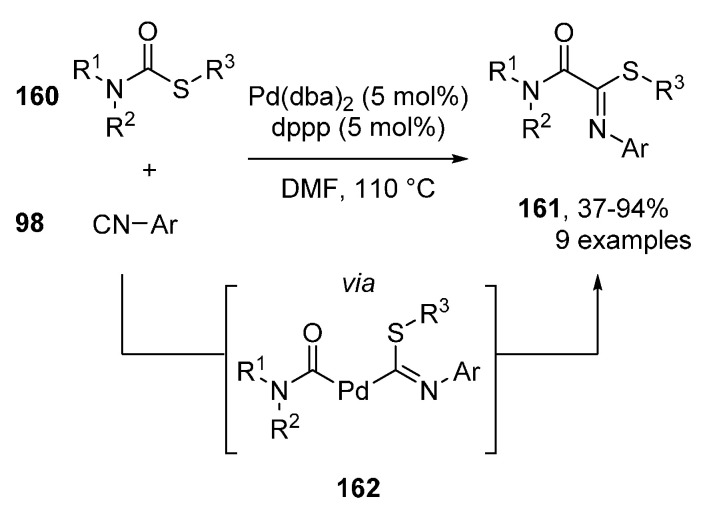
Pd-catalyzed isocyanide insertion into thiocarbamates.

Homologation of thiocarbamates **160** with isocyanides was also reported. [84] Treatment of thiocarbamates with a palladium catalyst and aromatic isocyanides results in the transient palladium complex **162** as an active intermediate. In silico experiments indicate the isocyanide insertion proceeds in the labile Pd-S bond. Subsequent reductive elimination affords the thioimidates **161** (Scheme 45). The isocyanide scope was not studied in detail and only 2,6-disubstituted aromatic isocyanides **98** were reported. Although the reaction tolerates different substituents on the thiocarbamate nitrogen, only *S*-arylated thiocarbamates were employed. Additionally, selenocarbamate analogues of **160** displayed similar reactivity, affording the corresponding products in 41–64%.

**Scheme 46:**
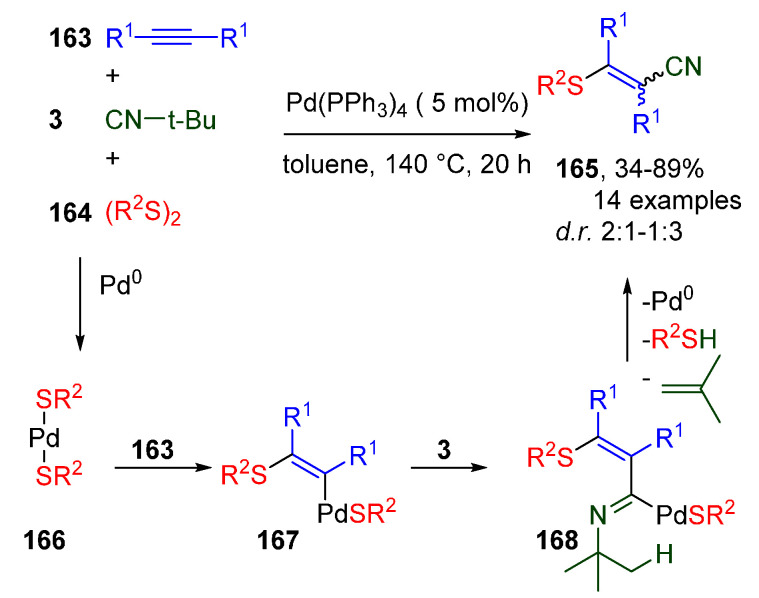
β-thiolated acrylonitrile synthesis from disulfide, *t*-butyl isocyanide, and internal alkynes.

Another unconventional Liebeskind-Srogl-type activation proceeds via the oxidative addition of disulfide **164** to the active Pd^0^ catalyst, followed by thiopalladation of the internal alkyne **163** (Scheme 46) [85]. The resulting vinylpalladium species **167** can undergo isocyanide insertion, after which δ-hydride elimination of the intermediate imidoyl palladium species **168** affords the β-thiolated acrylonitrile **164**, isobutene, and a thiol, while regenerating the Pd^0^ catalyst. Interestingly, the thiol byproduct does not poison the catalyst towards further catalytic turnover. Unfortunately, the *E*/*Z*-stereoisomeric ratio appears to be under thermodynamic control, and affords configurational mixtures in all cases, typically slightly favoring *E*-configured acrylonitriles **165**. Only a single asymmetric internal alkyne was probed, affording total regioselectivity, but still similar mixtures of *E*/*Z*-stereoisomers.

### 2.5. Imidoylation of Palladium-Ligated Carbenes and Nitrenes

**Scheme 47:**
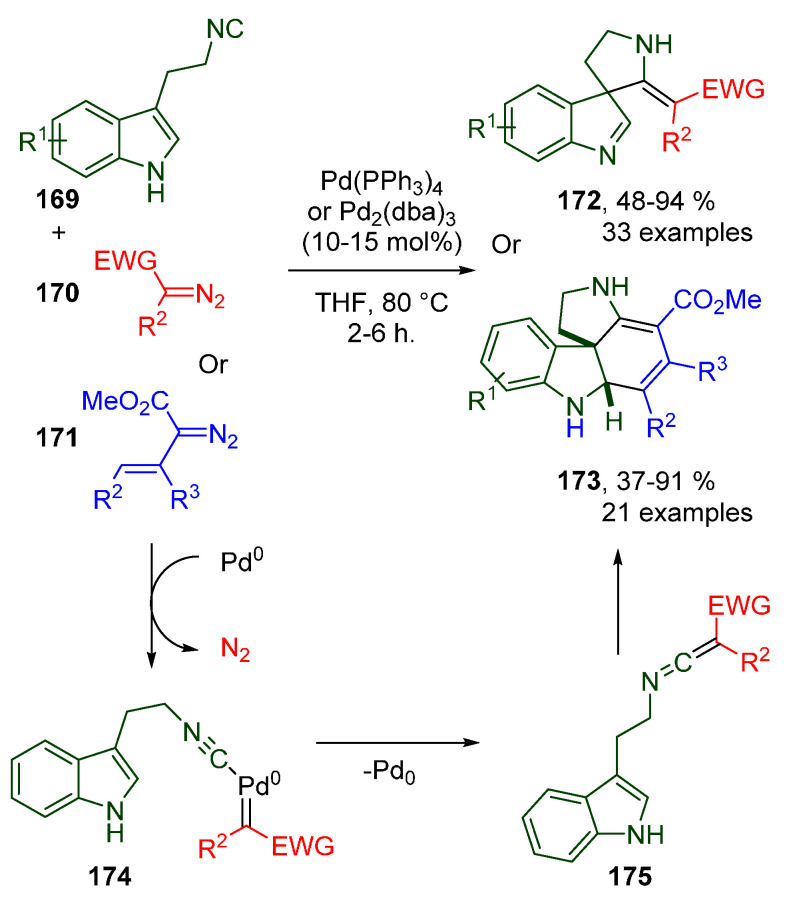
Pd-catalyzed carbene transfer from diazo compounds to tryptamine-derived isocyanides.

In 2020, the seminal work of Cai et al. [86] on carbene transfers to palladium isocyanide complexes was significantly extended to a cascade version. In this carbene transfer, which was reported by the group of Liu, the bifunctional nature of tryptamine-derived isocyanides **169** was utilized in an elegant fashion (Scheme 47) [87]. The authors proposed a palladium-catalyzed carbene transfer of diazoacetate **170** to the isocyanide, affording the corresponding ketenimine **175**, which readily undergoes dearomatization-spirocyclization upon nucleophilic attack of the indole C3-position, affording indolenines **172**. Further intramolecular annulation pathways are available if functionalized diazoacetates **171** are used, affording tetracyclic indolines **173** in moderate to excellent yields. It should be noted that the products **172** were readily derivatized in a enantioselective manner through asymmetric Mannich-type cyclization with chiral phosphoric acids.

**Scheme 48:**
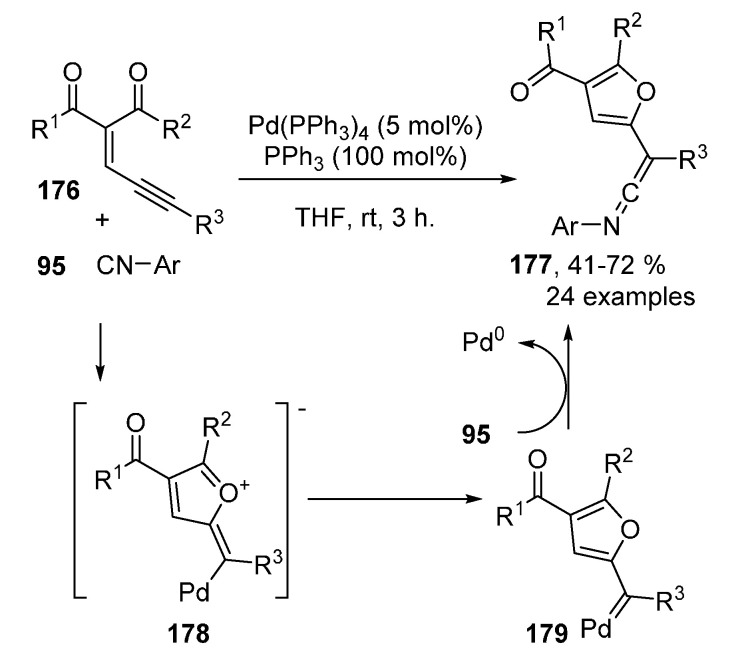
Pd-catalyzed carbene transfer resulting from 5-*exo*-dig cyclization to isocyanides.

Further advancements include generating the intermediate palladium carbene complexes in other ways than via diazo compounds. The palladium carbene complex **179** was obtained via a 5-*exo*-dig cyclization of conjugated enynones **176**, presumably via the zwitterionic intermediate **178** (Scheme 48) [88]. The carbene transfer to the isocyanides **95** proceeds smoothly, affording the polyfunctionalized furans **177** in isolated yields of 41–72%. In this case, only aromatic 2,6-disubstituted isocyanides were converted.

**Scheme 49:**
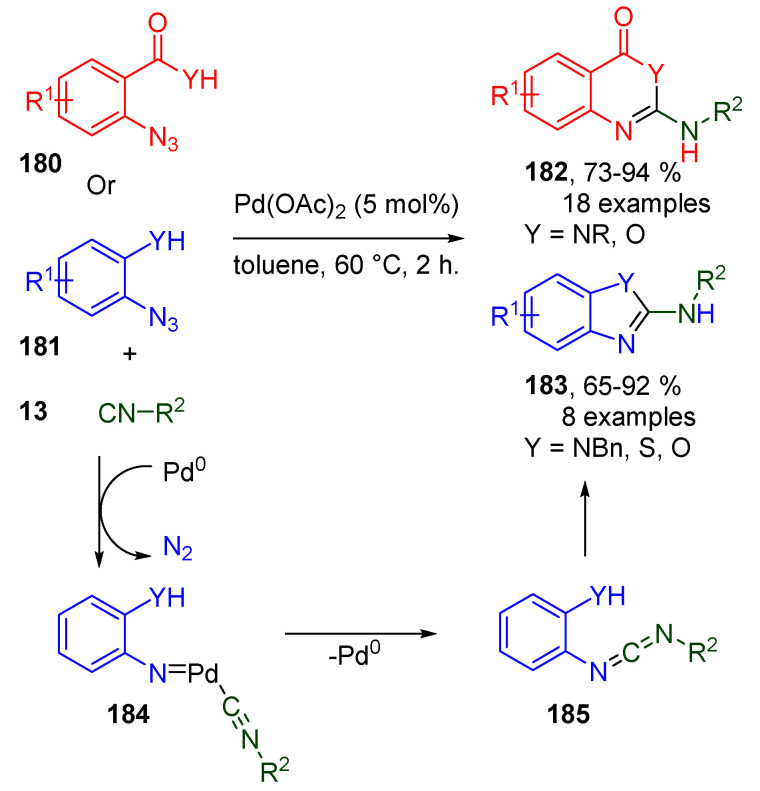
Pd-catalyzed nitrene transfer from bifunctional arylazides to isocyanides.

Analogously, starting from organic azides, the palladium-catalyzed transfers of nitrenes to isocyanide functionalities can be achieved. In a recent example, Sawant et al. reported a ligand-free substrate-dependent synthesis of bicyclic systems from bifunctional arylazide substrates [89,90]. Molecular nitrogen is readily extruded from the azide substrates to generate a palladium-bound nitrene intermediate **184**, which is subsequently transferred to the isocyanide to afford the short-lived carbodiimide **185**, which readily undergoes intramolecular addition (Scheme 49). The substrate scope includes both 2-azidobenzoic acids and amides **180**, which afford the corresponding benzoxazinones and quinazolinones **182** in good yields. Additionally, 2-azidoanilines and 2-azido(thio)phenols can also be converted to the corresponding 2-aminobenzimidazoles, -oxazoles, and -thiazoles **183** in good yields. These syntheses were only attempted with *tert*-butyl and cyclohexyl isocyanide. Extensive DFT calculations indicate the rate-determining step is the loss of nitrogen from the azide-palladium complex. The resulting nitrene is transferred to the isocyanide, and the resulting carbodiimide undergoes cyclization to afford the heterocycles **182** and **183**. In the same year, Ding et al. reported multiple one-pot post-imidoylation cascade transformations based on similar starting materials [91].

**Scheme 50:**
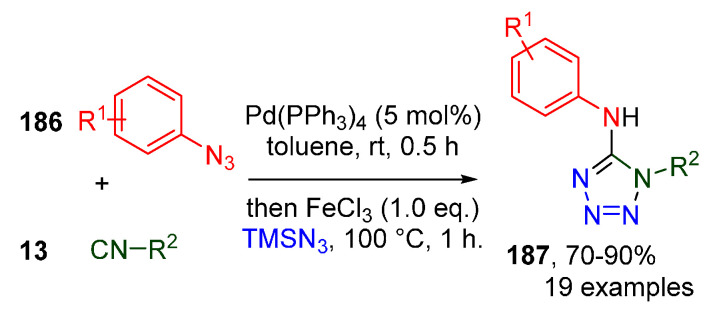
Pd-catalyzed nitrene transfer from azide to isocyanides and subsequent [3 + 2] cycloaddition.

This group further expanded the follow-up chemistry in a one-pot process [92]. The initial nitrene transfer of azide precursors **186** to isocyanides **5** under Pd catalysis proceeds in yields up to 90%. Post-coupling with trimethylsilyl azide in the presence of FeCl_3_ affords the medicinally valuable 5-amino-*1H*-tetrazoles **187** (Scheme 50)**.** This transformation tolerates tertiary, secondary, and aromatic isocyanides affording the corresponding aminotetrazole **187** with full regioselectivity in good to excellent yields. However, only starting materials are isolated if aliphatic azides are employed.

**Scheme 51:**
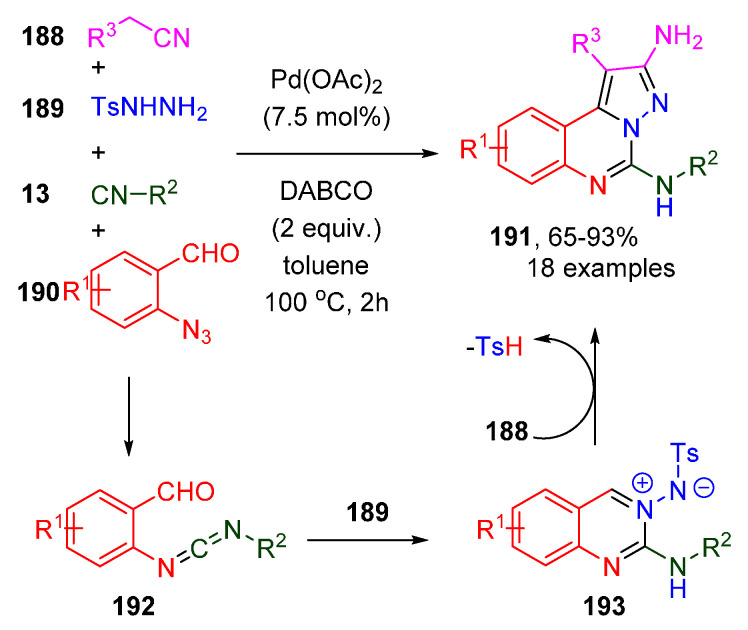
4-Component reaction towards pyrazolo[1,5-c]quinazolines, initiated by nitrene transfer from 2-azidobenzaldehydes to isocyanides.

An interesting 4-CR, initiated by palladium-catalyzed nitrene transfer of 2-azidobenzaldehydes **190** to isocyanides selectively affords carbodiimide **192** (Scheme 51) [93]. The authors postulate biscondensation with *N*-tosylhydrazine **189** forms the zwitterionic quinazolines **193**. Subsequent cyclcondensation with primary nitriles **188** yields tricyclic pyrazolo[1,5-c]quinazolines **191** bearing a free amine moiety. These products exhibit high EGFR inhibition, and are thus of interest as potential anti-cancer agents.

**Scheme 52:**
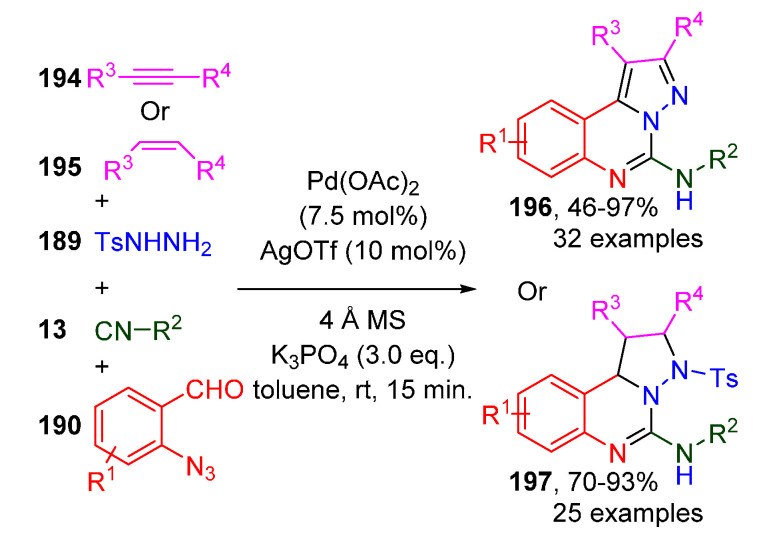
4-Component reaction towards pyrazolo[1,5-c]quinazolines, initiated by nitrene transfer from 2- azidobenzaldehydes to isocyanides involving olefins.

Additionally, the formed zwitterionic quinazolines **193** can undergo reaction with other partners than nitrile. A silver-catalyzed annulation with alkynes **194** cleanly affords the tricyclic pyrazoloquinazolines **196** under remarkably mild conditions (Scheme 52) [94]. The expulsion of *p*-toluenesulfinic acid occurs upon aromatizating formation of product **195**. If alkenes **195** are used in this 4-CR, the coupling generates the corresponding fused *N*-tosyl-tetrahydro pyrazoloquinazolines **197**.

### 2.6. Miscellaneous Pd^0^-Catalyzed Isocyanide Insertions

**Scheme 53:**
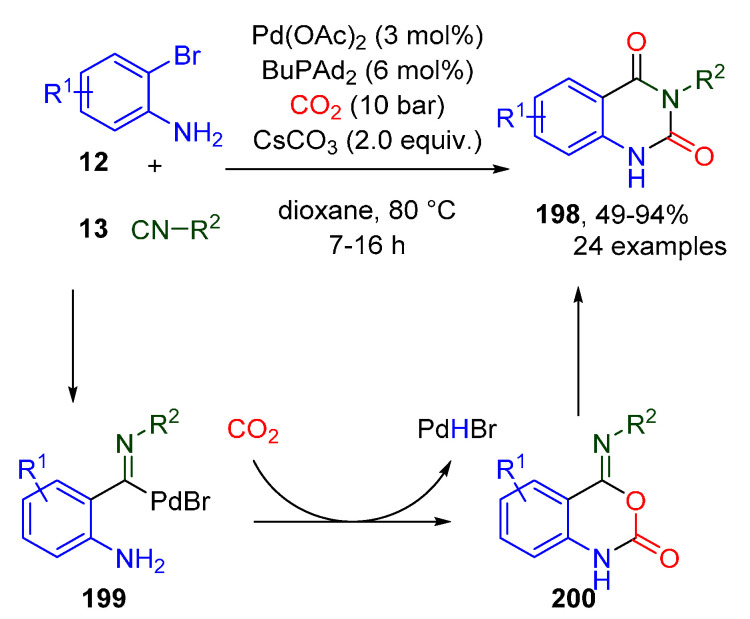
Sequential insertion of isocyanides and carbon dioxide in 2-bromoanilines.

Our group developed a quite uncommon sequential insertion of isocyanides and carbon dioxide [95]. This reaction proceeds via the Pd^0^ catalyzed imidoylation of *o*-bromoaniline **12** to form imidoyl palladium complex **199** (Scheme 53). A relatively high CO_2_ pressure is required to prevent this intermediate from undergoing a second isocyanide insertion (see **81**, Scheme 26), and favoring carboxylation. Subsequent reductive elimination affords benzoxazinones **200**, which undergo a Mazurkiewicz-Ganesan-type rearrangement under the optimized reaction conditions, resulting in the thermodynamically more stable substituted quinazolinediones **198**. This multicomponent cross-coupling tolerates tertiary, secondary, and primary, (including benzylic) isocyanides. The yield of the quinazolinediones **198** does not depend on the type of isocyanide employed, but appears to be correlated to the electron density of the used aniline **12**. A similar methodology was simultaneously reported by Ji et al. [96] Recently, palladium immobilized on an aminated graphene oxide layer was utilized as a heterogeneous catalyst in this imidoylative MCR. This catalyst requires 2-iodoanilines but the reaction can be executed at only 1 bar of CO_2_ [97].

**Scheme 54:**
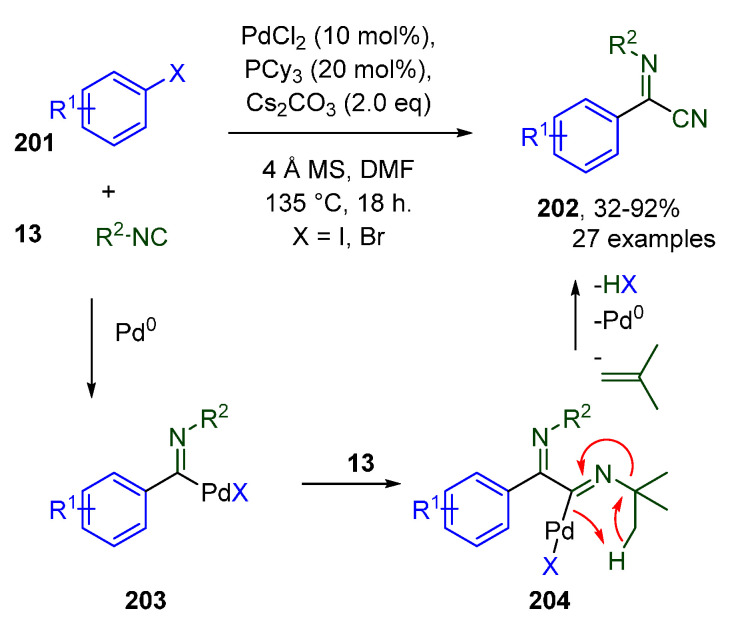
Synthesis of imidoylcyanides via reaction of aryl halides and *tert*-butyl isocyanide.

Another uncommon, but highly interesting formal cyanoimidoylation of aryl halides affords α-iminonitriles **202** through sequential isocyanide insertion/de-*tert*-butylation (Scheme 54) [98]. The concomitantly formed H-Pd-X can expel HX via reductive elimination, regenerating Pd^0^. The reaction conditions are remarkably tolerant of various substituents on the aryl halide **201**, including nucleophiles and heterocycles. However, introduction of an *o*-substitution significantly diminishes the yield. Again, the tertiary isocyanide functionality is required, as the reaction fails with other isocyanide substituents.

**Scheme 55:**
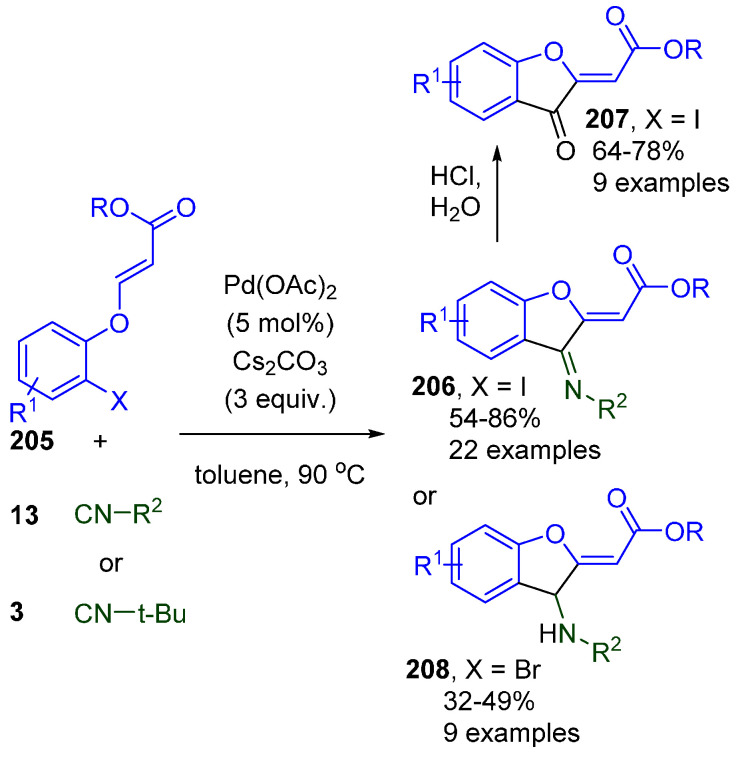
Imidoylative Heck-cyclizations (X = I, Br).

Over the past couple of years, alkene insertions involving isocyanide insertion have become an increasingly popular method to generate chemical complexity. Currently, the only report of a true imidoylative Heck-reaction is shown in Scheme 55, effectively inserting the isocyanide between the aryl halide and the activated alkene, in a tunable synthesis of iminoaurones [99].

The iminoaurones **206** are generated in good to excellent yields. Satisfyingly, the catalytic system functions without any ligands, and tolerates various aromatic isocyanides, as even the unstable 2-naphtyl isocyanide afforded the corresponding iminoaurone in 54% yield. Post-coupling hydrolysis with aqueous HCl cleanly affords the corresponding aurones **207** with virtually no loss in yield. Curiously, when 2-bromophenoxyacrylates **205** were used, the main product was the dihydrobenzofuran **208**. The authors showed that β-hydride elimination leads to the HPdBr, which does not undergo reductive elimination as readily as its iodide analogue, but rather transfers a hydride to the newly formed imine. Dihydrobenzofurans **208** were isolated in moderate yields, and only tertiary isocyanides were tolerated.

Intramolecular alkene insertion preceding imidoylation are more common. Bringing alkenes in close proximity to an in situ formed aryl palladium species typically leads to 5-*exo*-dig carbopalladation. In the absence of β-hydrogens, no β-hydride elimination (Heck reaction) can occur, and the formed alkylpalladium intermediate **210** instead undergoes imidoylative cross-couplings. As two insertions selectively and sequentially occur, we treat these examples in this miscellaneous section.

**Scheme 56:**
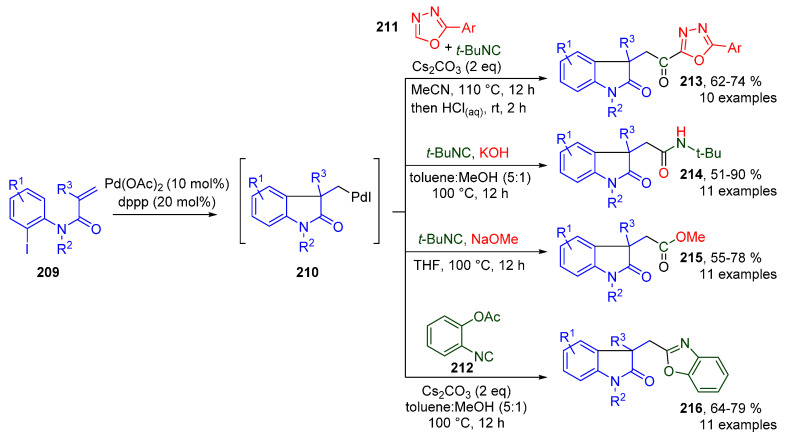
Imidoylative cross-couplings through alkene insertion/isocyanide insertion/reaction with nucleophiles.

The group of Zhu communicated several excellent examples of this reactivity, reporting a series of palladium-catalyzed reaction cascades. Intramolecular alkene insertion in *N*-(2-iodoaryl)acrylamides **209** delivers common intermediates **210**, which can undergo an isocyanide insertion (Scheme 56). Subsequent cross-coupling with oxadiazoles **211** involving C-H activation gave the ketones **213** after acid-mediated hydrolysis of the formed imine [100]. The authors further highlight the divergent nature of this cascade by changing the nucleophilic coupling partner. Utilization of KOH as a nucleophilic base affords the amides **214**, whereas the use of alkoxides leads to formation of the corresponding esters **215**. Additionally, substituting *tert*-butyl isocyanide for aromatic 2-acetoxyphenyl isocyanide **212** in which the acetylated phenol itself acts as a coupling partner, affording benzoxazoles **216** in good yields. This specific transformation might also result from of the base-mediated decomposition of isocyanide **212** to benzoxazole, in which case no formal isocyanide insertion occurs. Additionally, using the chiral DuanPhos ligand, the authors could also isolate analogs of amides **214** with up to 75% *ee*.

**Scheme 57:**
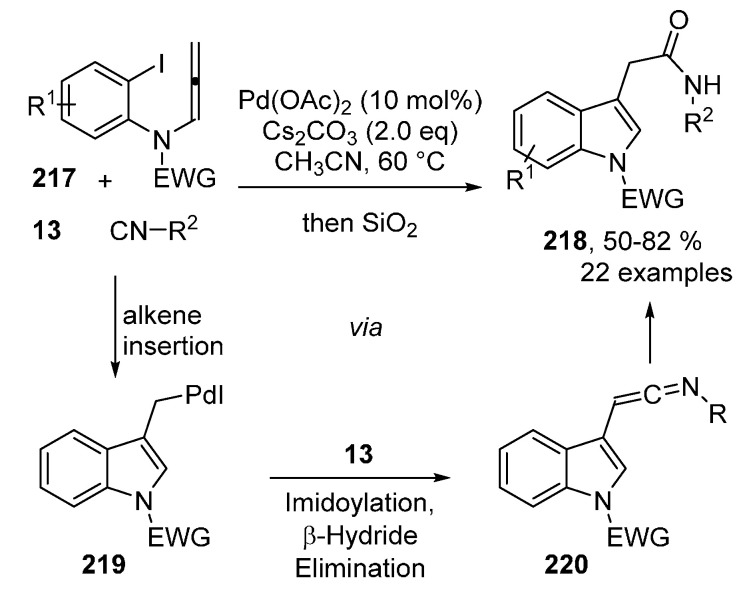
Imidoylative cross-couplings through alkene insertion/isocyanide insertion/reaction with nucleophiles.

Allenes can also be employed in these type of cascade transformations. The intramolecular carbopalladation of the distal double bond in *N*-protected *N*-allenyl-2-iodoanilines **217** proceeds selectively prior to isocyanide insertion [101]. The authors highlighted the divergent value of the synthetic method by trapping the intermediate ketenimine **220** with nucleophilic species such as water (Scheme 57). As a proof of concept, the intermediate **220** was also reacted by azides, alcohols, and amines to afford tetrazoles, esters, and amidines, respectively. Unfortunately, this transformation is only successful with tertiary isocyanides.

**Scheme 58:**
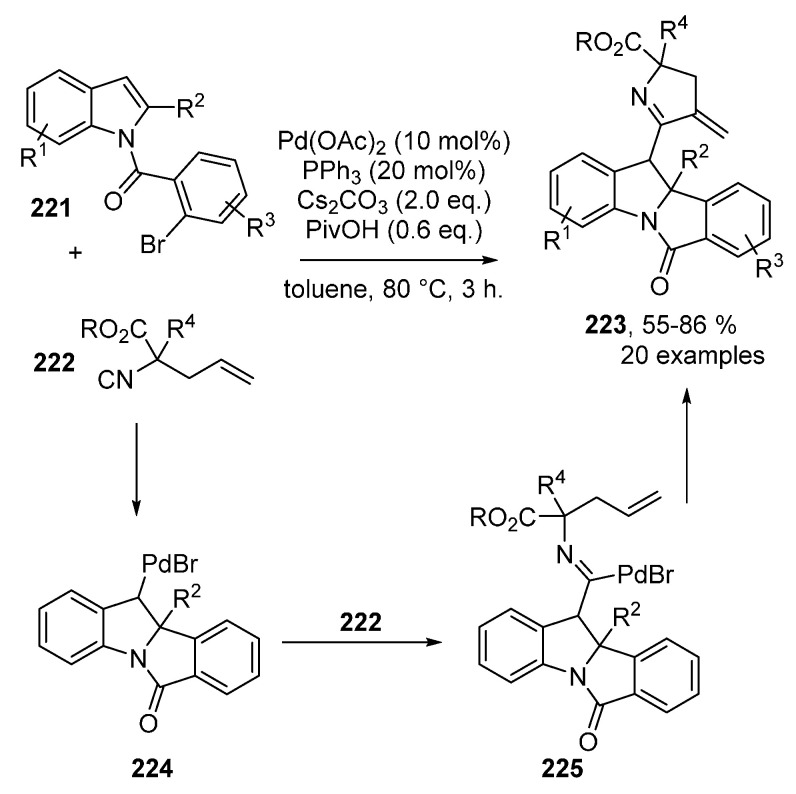
Dearomative imidoylation of indoles.

Very recently, Jiang et al. reported the first dearomative imidoylation of indoles [102], extending their earlier work with alkenyl isocyanides [103]. Combining readily available *N*-(2-bromobenzoyl)indoles **221** with these alkenyl isocyanides **222** gives a cascade reaction. The dearomative intramolecular carbopalladation outcompetes direct isocyanide insertion, forming the tetracyclic intermediate **224** (Scheme 58). Subsequent isocyanide insertion, and intramolecular Heck-type cyclization of the formed imidoyl-palladium species **225** affords indolines **223**. Unfortunately, isocyanides require slow addition over the course of the reaction, and the final Heck-cyclization is only successful if α-allyl isocyanoacetates are used. Longer or shorter tethers between the alkene and isocyanide moieties do not result in any product formation.

**Scheme 59:**
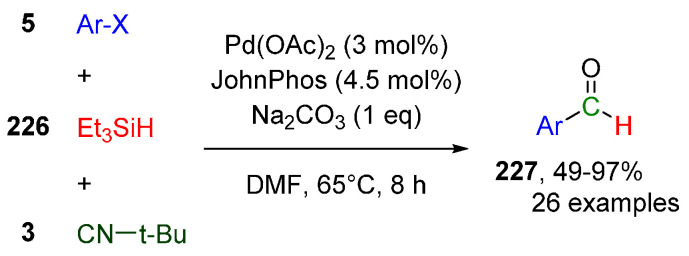
Reductive Pd-catalyzed isocyanide insertion using triethylsilane.

In 2014, the first reductive synthesis of aldehydes through a hydroimidoylation of aryl halides **5** was reported [104]. Although the reductive isocyanide insertion was only performed using *tert*-butyl isocyanide, the aryl halide input was varied extensively, affording aldehydes **227** in good to excellent yields, regardless of the steric or electronic influence of the arene substituents (Scheme 59). Triethylsilane **226** gives the best results, although other silanes also afford the target benzaldehydes. The use of silanes as reducing agents ensures the selective reduction of the intermediate imidoylpalladium species, but does not undergo reductive side reactions with the product aldimines. These aldimines undergo spontaneous hydrolysis upon aqueous workup.

**Scheme 60:**
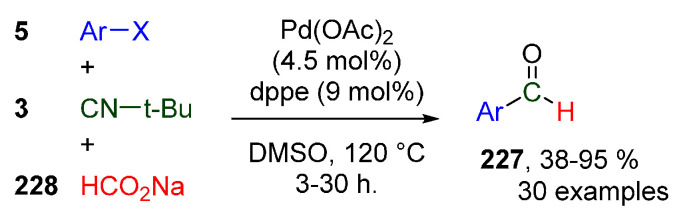
Reductive Pd-catalyzed isocyanide insertion using sodium formate.

A similar reductive isocyanide insertion generates benzaldehydes **227** from aryl iodides **5** (Scheme 60) [105]. In this case, sodium formate was found to be an effective reducing agent in polar solvents, allowing for the isolation of the product benzaldehydes in good yields, regardless of the aryl substitution pattern. Post-imidoylative hydrolysis of the aldimines occurred spontaneously upon extraction of the crude mixtures. The use of aryl bromides typically led to diminished yields, even under prolonged reaction times.

**Scheme 61:**
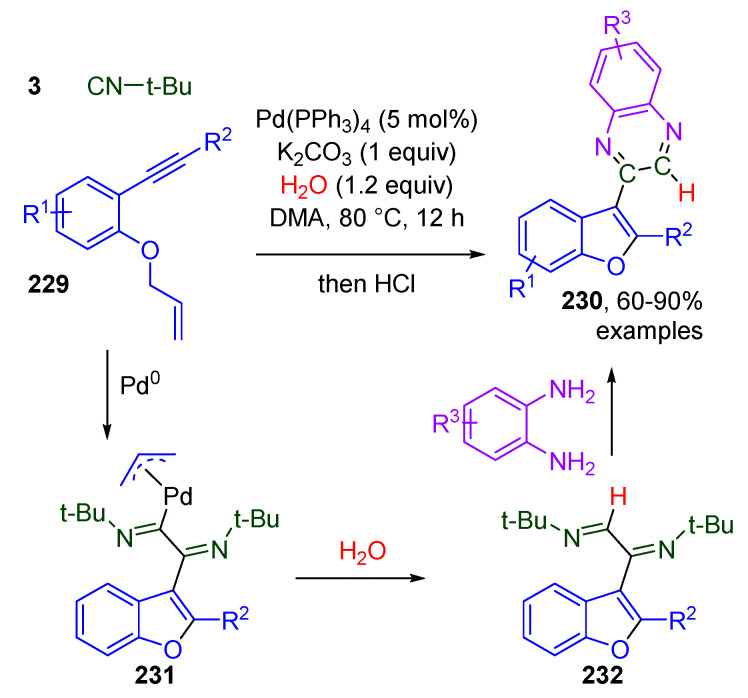
Imidoylative synthesis of 2-(benzofuran-3-yl)quinoxalines.

A divergent palladium-catalyzed synthesis of substituted 2-(benzofuran-3-yl)quinoxalines **230** has been shown to be quite effective [106]. The redox-neutral cross-coupling is initiated by the oxidative insertion of Pd^0^ into the allyl phenyl ether, which initiates a 5-*endo*-dig cyclization, furnishing the corresponding benzofuran palladium species (Scheme 61). For reasons that remain uninvestigated, this specific imidoylation proceeds through a double isocyanide insertion. Curiously, the resulting diimine complex **231** undergoes protodemetalation rather than hydroxylation. The authors did not investigate whether this process releases allyl alcohol or *N*-*tert*-butyl but-3-enamide as a byproduct. The formed glyoxal diimines **232** are subjected to sequential hydrolysis with aqueous HCl and biscondensation with phenylenediamines in a three-stage reaction. Additionally, the authors showed the versatility of this imidoylative synthesis by replacing the diamine condensation with several oxidative follow-up reactions.

**Scheme 62:**
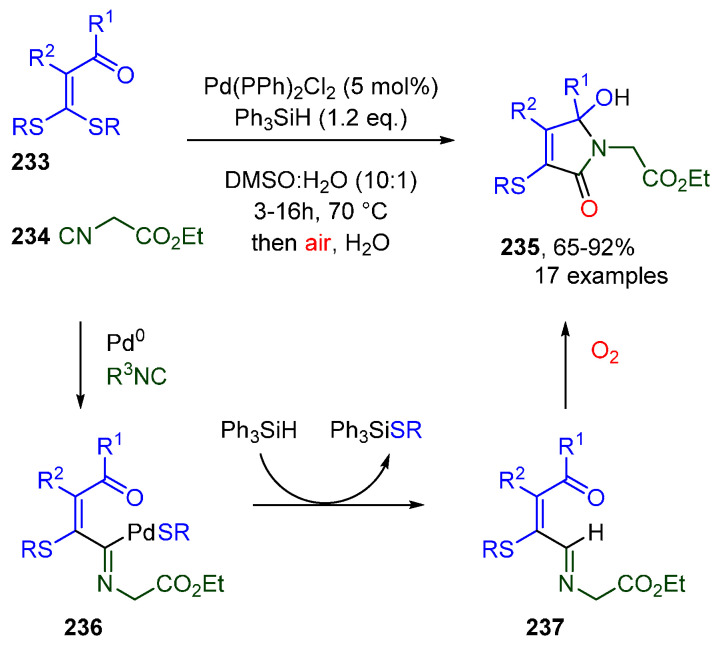
Imidolyative synthesis of 5-hydroxylated γ-lactams.

A novel imidoylative Liebeskind-Srogl-type reaction, with interesting mechanistic implications, was reported recently. [107] Oxidative insertion of Pd^0^ into the thioether **233**, and subsequent isocyanide insertion affords intermediate **236**, which is reduced by triphenylsilane, producing the enimine **237** (Scheme 62). The authors postulate that this enimine is the final product of the reaction, and only oxidizes to the oxaziridine upon aerobic workup, which rearranges to the lactams **235**. The role of the water in this process is not fully understood. The resulting 5-hydroxy-γ-lactams are isolated in good to excellent yields, although the use of isocyanoacetates **234** is critical in this transformation. The use of alkyl isocyanides led to uncyclized acrylamide analogs through a hydroxyimidoylation, whereas TosMIC is completely unreactive under the catalytic conditions.

## 3. Pd^II^-Catalyzed Isocyanide Insertions

### 3.1. Introduction

**Scheme 63:**
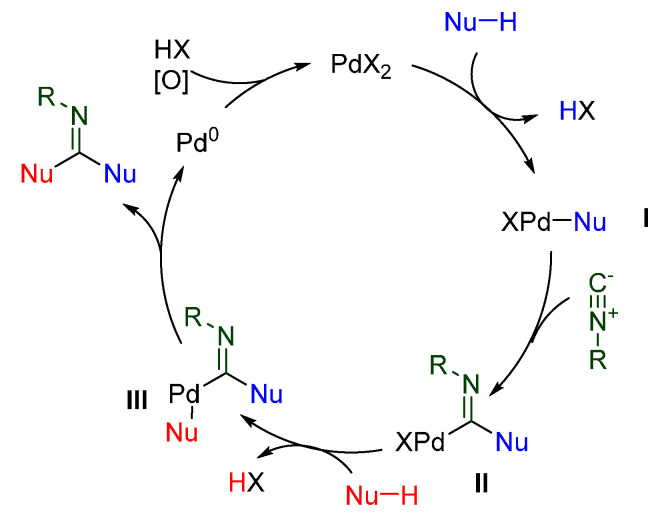
General catalytic cycle for cross coupling via oxidative isocyanide insertions.

The use of Pd^II^ as an active catalyst in cross-couplings involving isocyanide insertions largely concerns oxidative imidoylations. This allows the use of non-preactivated substrates. An oxidant is required to close the catalytic cycle. Conceptually, these reactions can be considered much more environmentally-friendly, although in reality this may depend on the reoxidizing agent used. In general, for scale-up purposes, oxidants which are cheap, low in mass, only produce benign by-products that can be safely disposed are preferred, such as O_2_, H_2_O_2_, NaOCl, AcOOH, and *t*-BuOOH [108,109]. A generalized catalytic cycle is characterized by a nucleophilic displacement of an anionic ligand from the active Pd^II^ catalyst, generating complex **I** (Scheme 63). Subsequent 1,1-migratory insertion of the isocyanide affords the imidoyl palladium species **II**, which is similar to the mechanism described in Scheme 1. Intermediate **II** can undergo a second nucleophilic displacement, upon which the intermediate **III** affords the reaction product upon reductive elimination. Finally, an oxidizing agent is needed to regenerate the active Pd^II^ catalyst. It should be noted that nucleophile has to be interpreted in the most general meaning, i.e., C-H activation can also give access to a Pd-C covalent bond without altering the oxidation state, still allowing for the formation of intermediates **I** and **III**.

### 3.2. Pd^II^-Catalyzed Intermolecular Oxidative Isocyanide Insertions

**Scheme 64:**
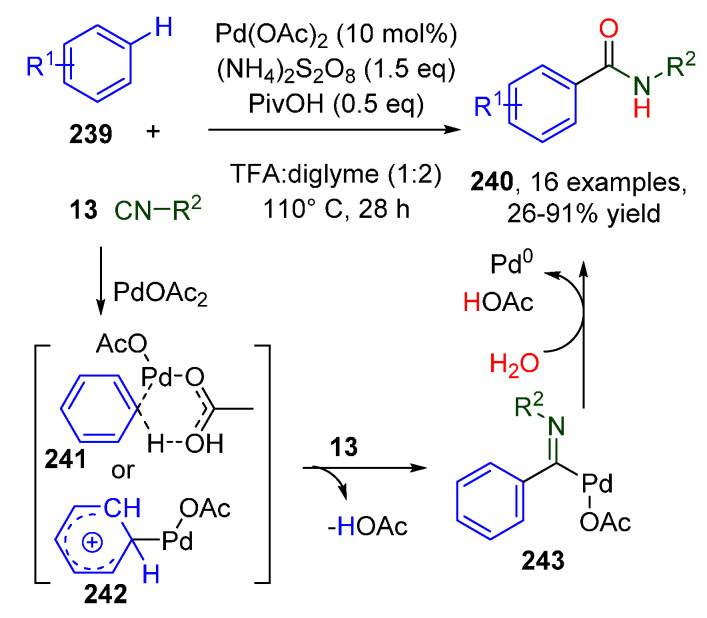
Oxidative C-H functionalization of arenes with isocyanides and water. (X = OAc).

A direct synthesis of benzamides **240** from arenes **239**, isocyanides **13** and water was recently reported by Akbari et al. (Scheme 64) [110]. This C-H functionalization takes place on the most electron-rich position, implying an electrophilic aromatic substitution with the active palladium(II) species for C-H activation, i.e., through intermediate **242** rather than **241**, although the mechanism has not been fully elucidated. The authors believe that imidoylation of the afforded aryl palladium intermediate generates **243**, which yields benzamides **240** upon reaction with water. (NH_4_)_2_S_2_O_8_ was used as the terminal oxidant. Peroxydisulfates are cheap oxidants produced on a large scale, mainly used to initiate polymerization and to etch metal [111]. This oxidative amidation exhibits a remarkable tolerance with regards to the isocyanide scope, as aliphatic, benzylic, aromatic, and α-acidic isocyanides all afford the benzamides **240** in good to excellent yields.

**Scheme 65:**
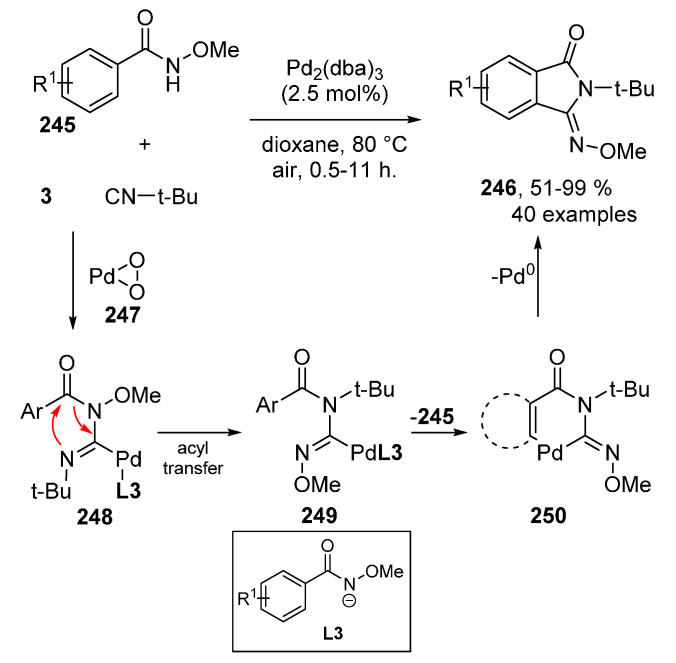
Oxidative C-H functionalization of *N*-methoxybenzamides with isocyanides providing iminophthalimides.

The group of Yu reported an efficient C(sp^2^)-H imidoylation using *N*-methoxybenzamides **245** as a substrate and a directing group for the rate-determining C-H activation [112]. The active catalyst **247** was later proposed [113] to deprotonate the hydroxybenzamide, forming palladium carboximidamide intermediate **248**. The authors found the rearrangement is not a post-imidoylative Mazurkiewicz-type process, but rather occurs via an acyl-migration in this intermediate **248** (Scheme 65). This affords the palladium species **249**, which then undergoes C-H activation to **250** and reductive elimination. This reaction tolerates many different substitutions on the benzamide, and also affords the corresponding fused iminophthalimides **246** when heterocyclic *N*-methoxyamides are employed. However, only *t*-BuNC was used as an isocyanide input. The reaction still affords iminophthalimides **246** when the benzamide ring is substituted with other potential directing groups, known to facilitate C-H functionalization. The products **246** are susceptible to catalytic hydrogenation, affording isoindolin-1-ones, effectively using isocyanides as a formal CH_2_-donor.

**Scheme 66:**
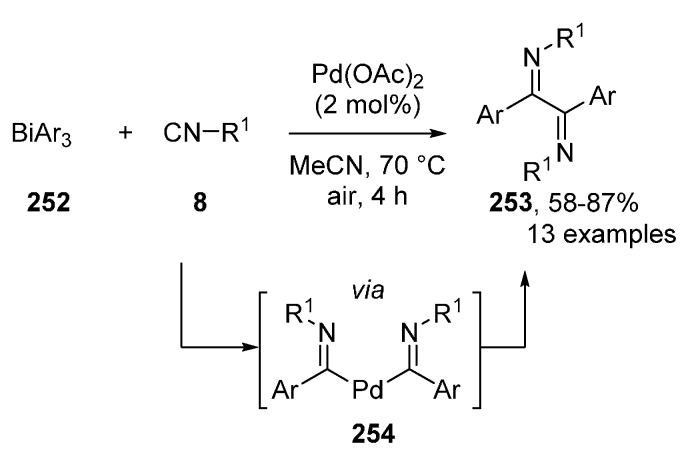
Double isocyanide insertion using triaryl bismuth reactants.

An example of a selective formal double isocyanide insertion is shown in Scheme 66, using an unconventional triarylbismuth reactant **252** as a transmetallating agent. The authors propose that selective formation of the bisimine product **253** proceeds via Pd species **254** [114]. The reaction is significantly more effective under air atmosphere, implying reoxidation of Pd^0^ is more efficient with molecular oxygen than with Bi(OAc)_3_. Surprisingly, cross-coupling with arylbismuths **252** tolerates various isocyanides, inserting aliphatic, benzylic, and even aromatic isocyanides in an effective manner.

**Scheme 67:**
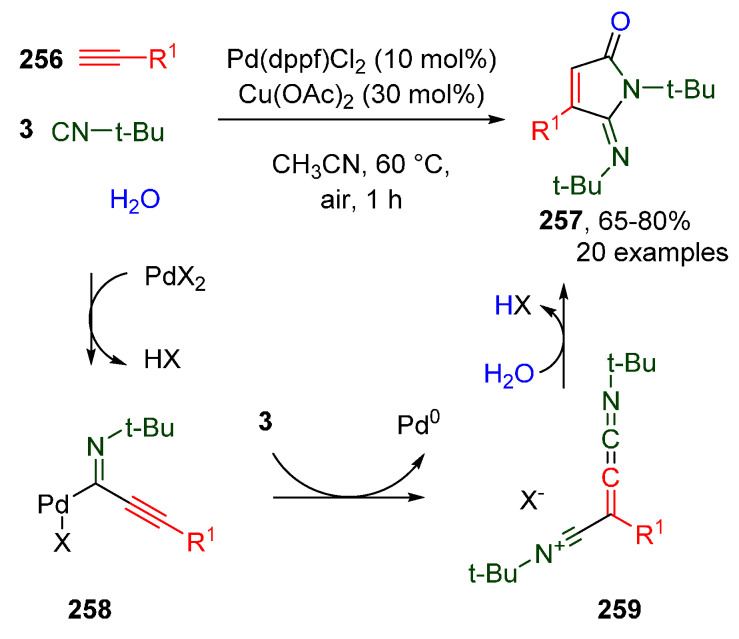
Iminomaleimides from two isocyanides, water, and terminal alkynes (X = OAc, Cl).

The oxidative Pd-catalyzed synthesis of iminomaleimides **257** from alkynes, *tert*-butyl isocyanide and one equivalent of water has been reported. Electronically and sterically diverse alkynes **256** are compatible with the devised methodology, although no internal alkynes were included [115]. The authors propose copper acetate acts solely as an oxidant, although the possible catalytic activity towards the proposed intermediate **258** was not investigated in more detail. As copper is added in a catalytic amount, air functions as the terminal oxidant. External attack of a second isocyanide is hypothesized to form the cationic intermediate **259**. While this transformation was successful with tertiary isocyanides, all other isocyanides failed to give the corresponding *N*-substituted analogs of **257** (Scheme 67).

**Scheme 68:**
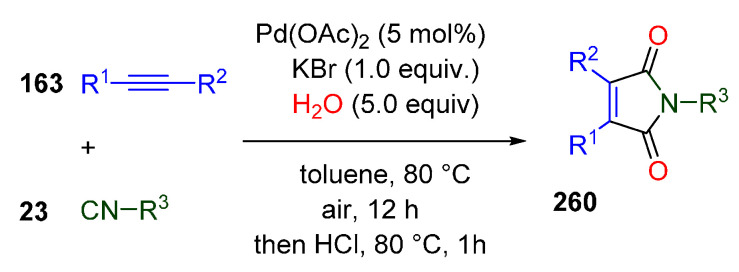
Maleimides from two isocyanides, water, and alkynes.

In the same year, it was reported that maleimides **260** can be synthesized via a similar oxidative double imidoylation of internal alkynes **163** affording the corresponding maleimides **260** in good yields (Scheme 68) [116]. Air acts as a terminal oxidant. In this particular reaction, various substituents on the alkyne are well tolerated, as even ethynamines or phenylpropiolates display high compatibility. Due to the hydrolytic cleavage of the imine, symmetrical maleimides **260** are always obtained, and no investigations were made into the regioselectivity of this coupling. Finally, the compatibility with tertiary, secondary, and primary isocyanides is high, but no further investigations into the isocyanide scope were performed. The same group later reported a similar transformation to phthalimides from *o*-bromobenzonitriles [117].

**Scheme 69:**
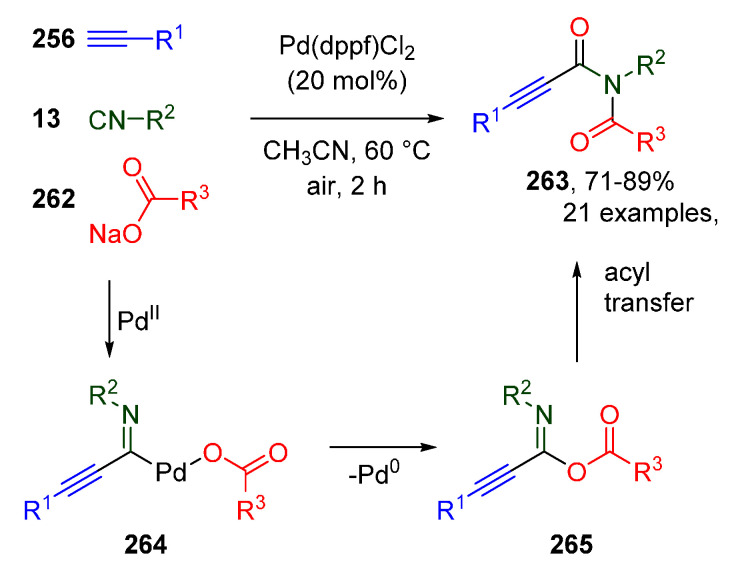
Imide synthesis from alkynes, isocyanides, and sodium carboxylates.

Follow-up chemistry using this reaction involved the trapping of the transient yniminyl palladium species with carboxylates (Scheme 69) [118]. Curiously, the utilization of carboxylates as coupling reactants rather than water does not lead to a nucleophilic attack of a second isocyanide, as observed above (Scheme 68). Instead, reductive elimination affords the *O*-acylimidate intermediate **265**, which can undergo a Mumm-type intramolecular acyl transfer to generate the product imides **263**. This transformation is highly compatible with primary and aromatic alkynes, as well as with benzylic and aromatic isocyanides. Although this method is highly compatible with benzylic or aromatic isocyanides, this specific transformation did not afford any product when tertiary or secondary aliphatic isocyanides were used. Additionally, in this case, air acts as the terminal oxidant reoxidizing Pd^0^.

**Scheme 70:**
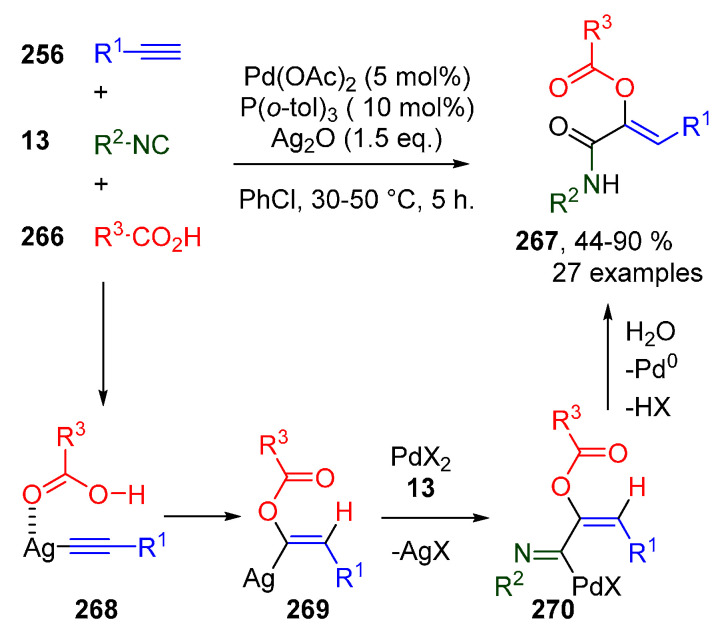
Oxidative Pd/Ag dual catalyzed synthesis of α-acyloxyacrylamides.

Another interesting Pd/Ag dual catalytic method was reported for the synthesis of enol esters **267** [119]. The reaction is initiated by the insertion of the terminal alkyne **256** into the silver carboxylate, generating intermediate **269** (Scheme 70). Transmetalation to Pd^II^, and subsequent hydroxyimidoylation affords the product **267** in moderate to good yields. The formed Pd^0^ is readily reoxidized by the presence of stoichiometric Ag_2_O. Silver, therefore, plays a dual role here. This reaction is highly compatible with aliphatic and aromatic alkynes; however, when α-acidic isocyanoacetates are employed, no product is observed.

**Scheme 71:**
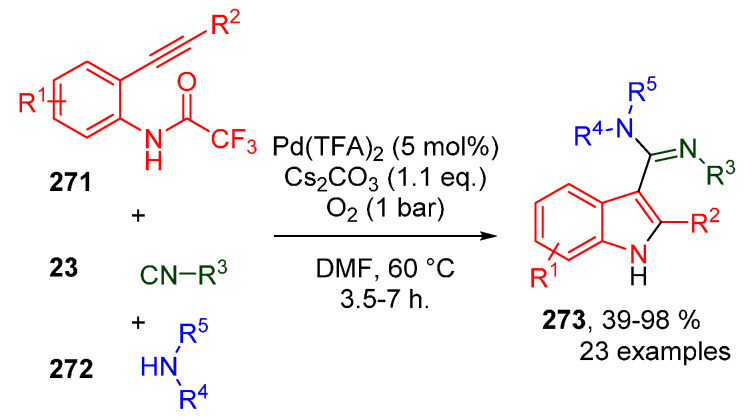
Oxidative Pd-catalyzed 5-*endo*-dig cyclization followed by aminoimidoylation.

In 2013, Zhu et al. performed indole-3-carboximidamides synthesis from *N*-trifluoracetyl-2-alkynylanilines **271** via a 5-*endo*-dig cyclization, and subsequent aminoimidoylation [120]. O_2_ was used as the terminal oxidant. This oxidative cyclization is compatible with various aromatic substituents, regardless of the electronic nature (Scheme 71). Both secondary and primary amines **272** (R^5^ = H) are also readily converted to the corresponding amidine, but the use of aromatic amines leads to reduced yields of the corresponding products **273**. Although secondary and primary isocyanides are tolerated, the yields decrease dramatically when these non-tertiary isocyanides are used (39–45%). It is likely that aqueous workup leads to rapid hydrolysis of the initial *N*-trifluoroacetylated indole product.

**Scheme 72:**
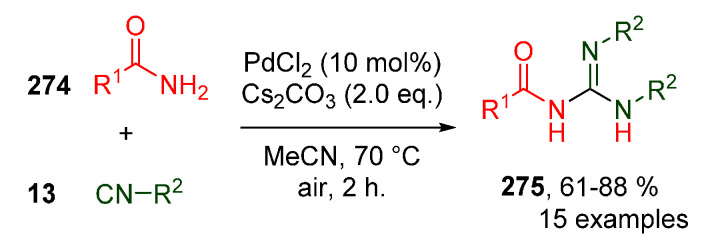
Oxidative Pd-catalyzed formation of *N*-acylguanidines using amides and isocyanides.

Additionally, an oxidative aminoiminoylation of primary amides **274** affords the substituted *N*-acylguanidines **275** (Scheme 72) [121]. This reaction was only tried with *tert*-butyl and cyclohexyl isocyanides, and proceeds rapidly, using air as the (re)oxidant. An equivalent of amine is formed by the known [122] oxidation of isocyanide to isocyanate, and its subsequent hydrolysis, effectively using the isocyanide as a dual reactant.

**Scheme 73:**
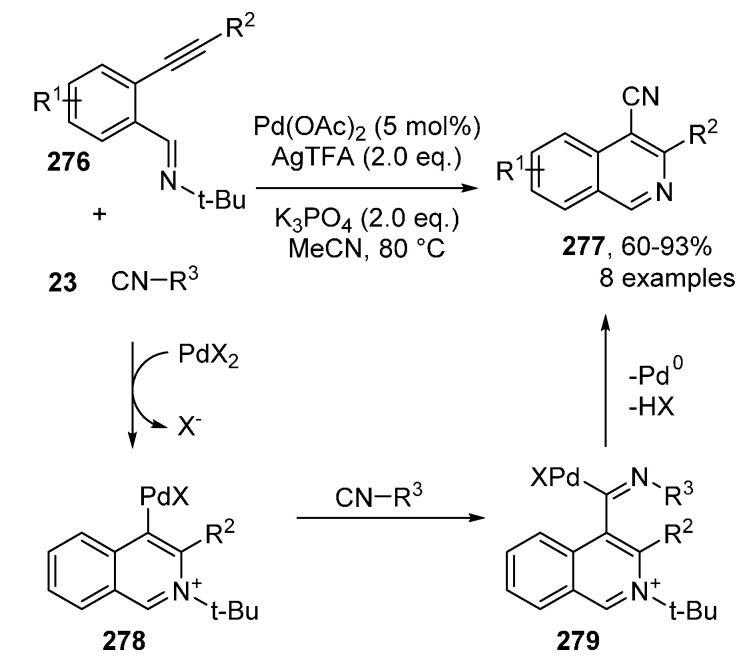
3-Cyanoisoquinoline synthesis via a 6-*endo*-dig cyclization/isocyanide insertion/double dealkylation cascade.

A different approach to the utilization of isocyanide as dual reactant is shown in Scheme 73. The cationic intermediate **278** is generated via a 6-*endo*-dig cyclization of *t*-butyl(2-alkynyl)benzaldimines, facilitating the *anti*-iminopalladation. Subsequent isocyanide insertion provides an imidoylpalladium species **279** that dealkylates affording the 4-cyanoisoquinoline **277**. Regeneration of the Pd^II^ catalyst occurs via oxidation with silver triflate. [123] Although cyanations with isocyanide as a CN-donor typically utilize *tert*-butyl isocyanide, due to its relatively facile dealkylation, this transformation also affords **277** if cyclohexyl isocyanide is inserted, albeit in somewhat lower yields. Additionally, if the solvent contains trace amounts of water, hydroxyimidoylation is preferred over dealkylation, affording the corresponding isoquinoline-4-carboxamides.

**Scheme 74:**
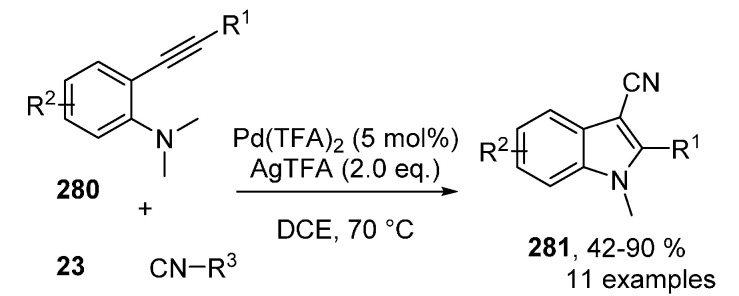
3-Cyano-*N*-methylindole synthesis through an anti-palladation/isocyanide insertion/dealkyation cascade.

Similarly, the formation of 3-cyano-*N*-methylindoles **281** occurs through a one-pot 5-*endo*-dig palladation/imidoylation/dealkylation/demethylation cascade from 2-alkynyl-*N*,*N*-dimethylanilines **280** and isocyanides (Scheme 74) [124]. A similar intermediate imidoyl palladium species as shown above (Scheme 73) is involved. Lack of a suitable coupling partner initiates R^3^ dealkylation. Here, too, dealkylation occurs if *tert*-butyl- or cyclohexyl isocyanide is used. The authors do not comment on the active demethylating agent.

**Scheme 75:**
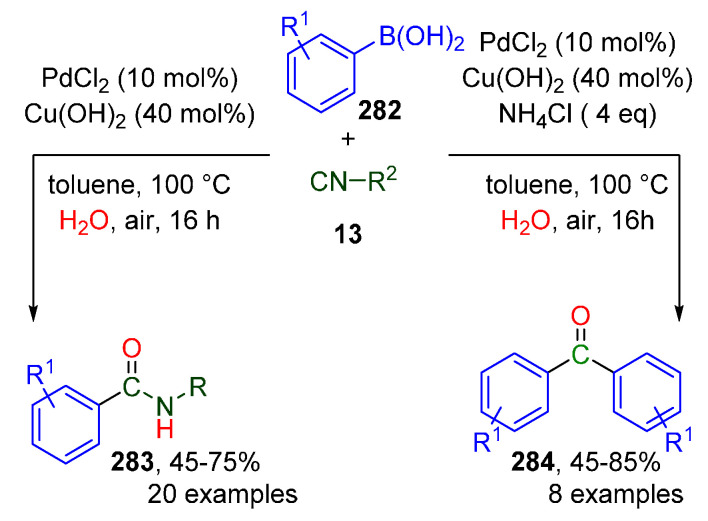
Pd/Cu co-catalyzed formation of benzamides or biaryl ketones.

Finally, intermolecular oxidative imidoylative cross-couplings can also be initiated by the addition of a boronic acid to an active Pd^II^ catalytic center. This effectively generates an aryl palladium intermediate (similar to Scheme 1) that readily undergoes 1,1-migratory insertion of an isocyanide. Such an imidoylation can take place under Pd/Cu co-catalysis, as evidenced by the divergent synthesis of benzamides **283** or biaryl ketones **284** in Scheme 75 [125]. Equimolar amounts of arylboronic acid **282** and isocyanides **13** afford the benzamides **283** where the intermediate imidoyl palladium species is quenched with ambient water. The authors propose that Cu(OH)_2_ serves to reoxidize the palladium catalyst, but no actual experimental study towards the role of copper in this mechanism was conducted. The isocyanide scope for this amidation is broad, tolerating aliphatic, as well as benzylic isocyanides. Aromatic isocyanides are also compatible with this amidation, even generating phenyl benzamide in 50% isolated yield, starting from the notoriously hard to handle phenyl isocyanide. On the other hand, when this reaction is performed with five equivalents of boronic acid under mild acidic conditions, an imidoylative Suzuki-type homocoupling is observed, affording biarylketones **284** after in situ hydrolysis. Although the isocyanide is cleaved off, the authors showed that this homocoupling is compatible with several isocyanides, mirroring the efficiency shown for the synthesis of benzamides **283**.

**Scheme 76:**
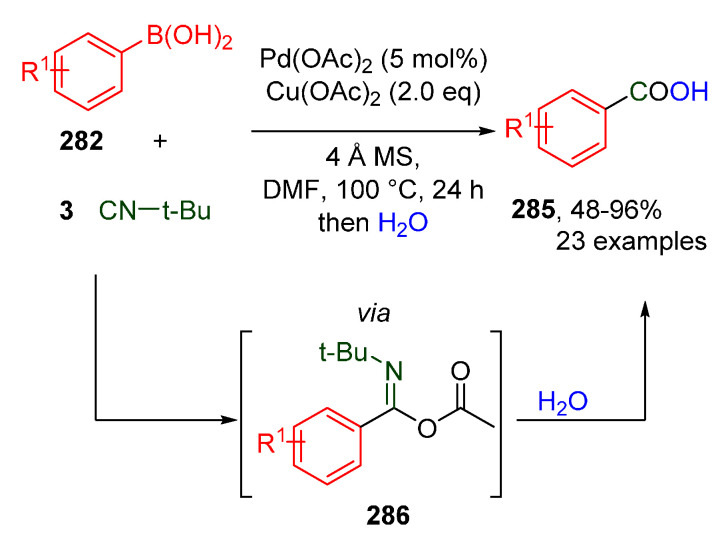
Synthesis of benzoic acids from arylboronic acids using isocyanides as a C_1_ building block.

Isocyanide insertion can be used in the acetoxyimidoylation of boronic acids **282**, utilizing *t*-BuNC as a C_1_ donor and subsequent hydrolysis (Scheme 76) [126]. The carboxylic acids **285** were formed in good yields, regardless of the electronic nature of the boronic acid. The reaction is sensitive to water during the cross-coupling, but hydrolysis of the formed intermediate **286** occurs upon quenching. The Cu(OAc)_2_ additive acts both as a stoichiometric oxidant to reform the active Pd(II) species, and as an acetate donor in the formation of **286**. No isocyanide variations were studied besides *tert*-butyl isocyanide.

**Scheme 77:**
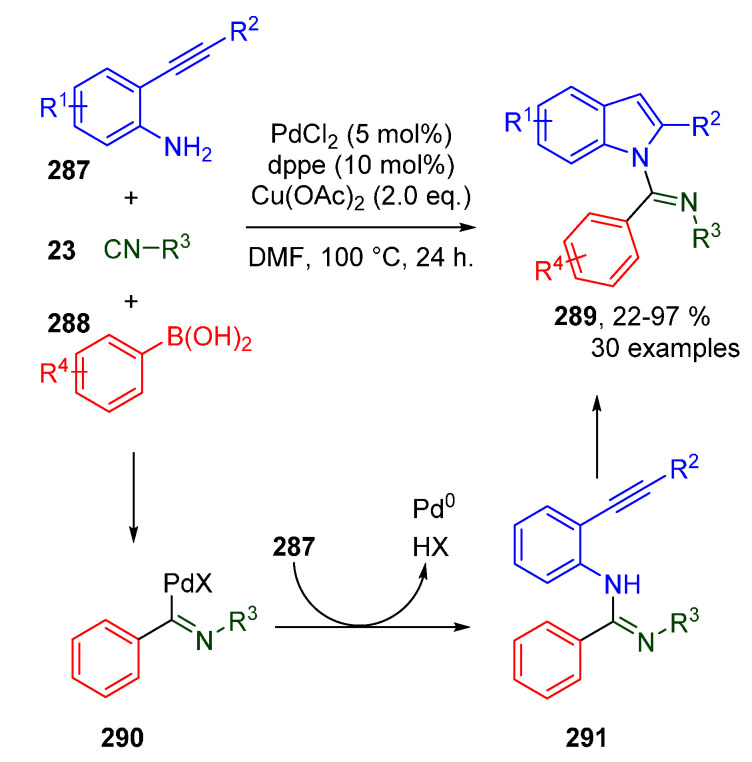
Pd-catalyzed Buchwald-Hartwig imidoylation followed by copper-mediated 5-*endo*-dig cyclization.

Pd-catalyzed imidoylative Buchwald-Hartwig reaction can also be combined with a cascade transition metal-catalyzed cyclization [127]. Transmetalation with arylboronic acids and 1,1-migratory insertion of the isocyanide affords the imidoyl palladium species **290**, which is subsequently trapped by the nucleophilic 2-alkynylaniline **296** (Scheme 77). The formed intermediate amidine **291** undergoes a copper-mediated 5-*endo*-dig cyclization to afford the substituted *N*-imidoylindoles **289** in varying yields. Copper also acts as a reoxidant for Pd^0^. The yields drop most noticeably when labile substituents are introduced on the boronic acid. The authors attempted a single deviation from tertiary isocyanides as a substrate, affording the corresponding product **289** in 53% when cyclohexyl isocyanide is used. Aromatic isocyanides are incompatible with this methodology.

### 3.3. Pd^II^-Catalyzed Intramolecular Oxidative Isocyanide Insertions

**Scheme 78:**
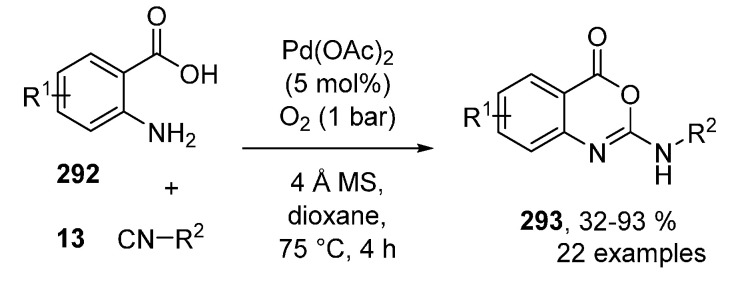
Oxidative isocyanide insertion into anthranilic acids.

Our group reported one of the first aerobic Pd-catalyzed formation of benzoxazinones **293** through the direct coupling of anthranilic acids **292** with isocyanides (Scheme 78) [128]. The reaction is compatible with several secondary and primary isocyanides, although utilization of non-tertiary isocyanides leads to a moderate yield of **293**, even at higher catalyst loadings (10 mol%).

**Scheme 79:**
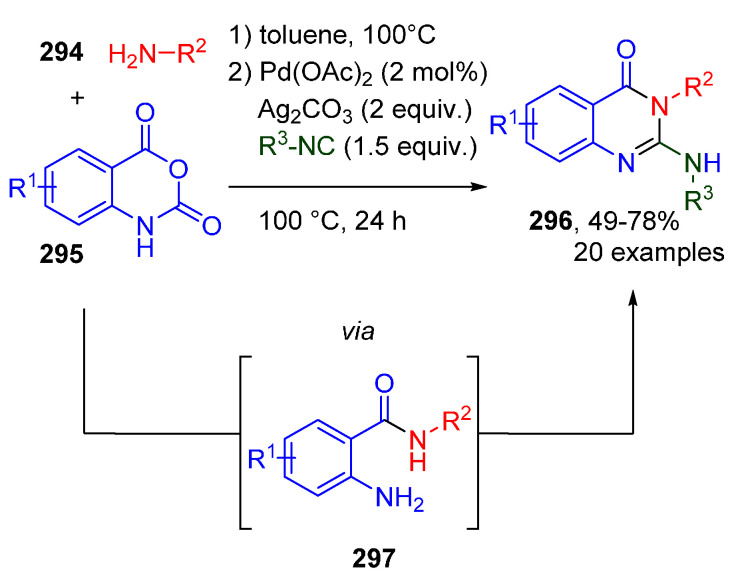
Oxidative isocyanide insertion into anthranilamides formed from isatoic anhydrides and amines.

Such bisnucleophilic substrates can also be generated in situ (Scheme 79). The condensation of isatoic anhydrides **295** with various aliphatic or aromatic amines **294** can be followed by addition of isocyanide and a palladium catalyst, without isolation of intermediate **297** or solvent switches, to afford 2-aminoquinazolinones **296** in a one-pot process [129]. For this palladium-catalyzed imidoylation, silver carbonate was found to be the optimal terminal oxidant. Unfortunately, while the reaction does appear to be fully compatible with tertiary and secondary isocyanides, the isocyanide scope was not extensively investigated.

**Scheme 80:**
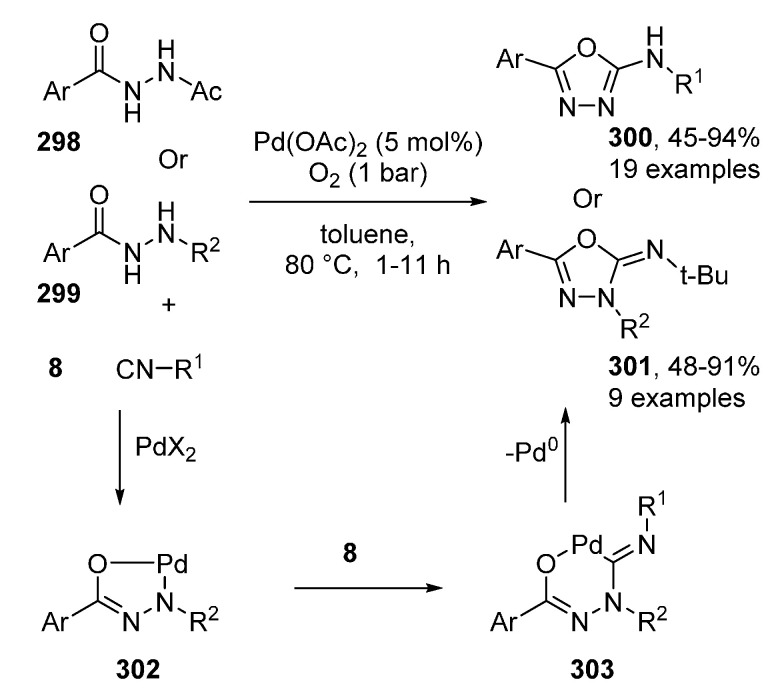
Oxidative isocyanide insertion into (*N*-acetylated) benzhydrazides.

An aerobic oxidative Pd-catalyzed imidoylative cross-coupling towards oxadiazoles **300** was performed by Fang et al. [130] Interestingly, the reaction was shown to be much more effective when *N*-acetylated benzhydrazides **298** were used, compared to N-H free hydrazides (**299**, R^2^ = H), although no explanation was offered. The acetylated oxadiazoles hydrolyze towards oxadiazoles **300** upon aqueous workup. Additionally, the incorporation of a substituent to the terminal nitrogen of the hydrazide **299** does not hamper the formation of the corresponding oxadiazol-2(3*H*)-imines **301** (Scheme 80), which have been reported as angiotensin II antagonists, and are typically difficult to prepare via other procedures [131]. Although the reaction is compatible with tertiary and secondary isocyanides, phenyl isocyanide undergoes rapid polymerization under the reaction conditions.

**Scheme 81:**
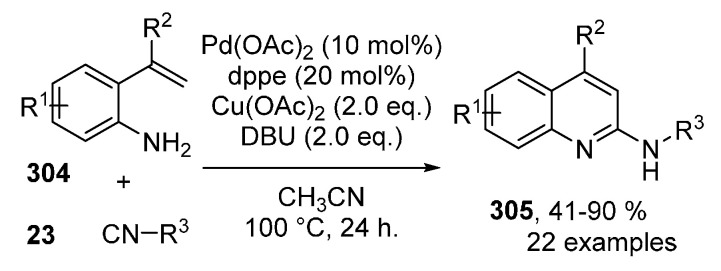
Oxidative isocyanide insertion into 2-aminostyrenes.

Similarly, electron-rich *o*-aminostyrenes **304** can also be subjected to oxidative isocyanide insertion, generating the 2-aminoquinoline scaffold (Scheme 81) [132]. The reaction is initiated by the coordination of the aniline to the active Pd^II^ complex. The aminoquinolines **305** are obtained in lower yields if electron-withdrawing aromatic substituents (R^1^) are present. Limited studies have been performed towards the tolerance for isocyanides. When primary or secondary aliphatic isocyanides are used, the 2-aminoquinolines **305** are obtained in moderate yields (48–61%). Again, the reaction conditions proved incompatible with any aromatic isocyanides.

**Scheme 82:**
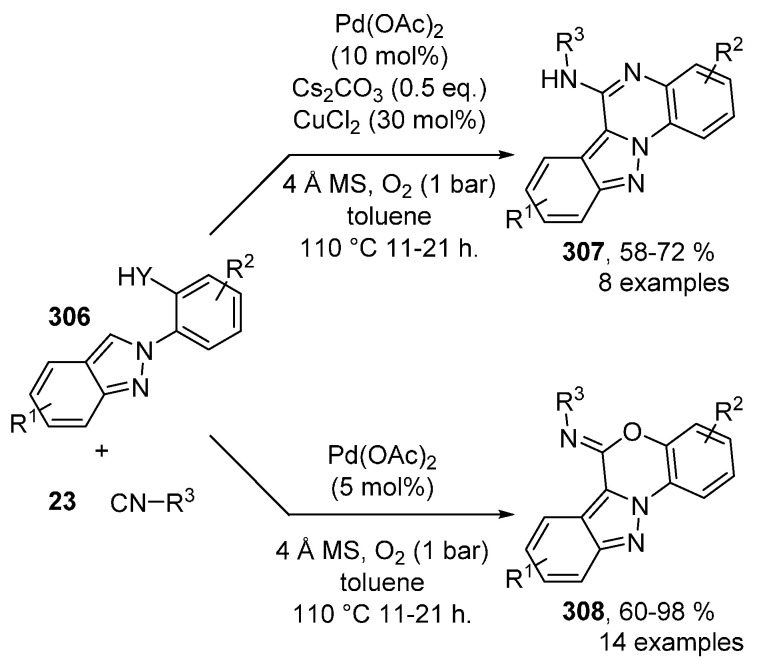
Oxidative imidoylation of 2-indazolylphenol and -aniline.

Treatment of electron-rich 2-indazol-2-yl phenols and -anilines were treated with isocyanides under aerobic oxidative conditions, in the presence of a palladium catalyst furnished the corresponding tetracyclic fused indazole scaffolds **307** (Scheme 82) [133]. While the synthesis of indazolo[2,3-a]quinoxalines **307** is relatively harsh, the use of 2-indazolylphenol (**306**, Y = O) affords the corresponding cycloimidate product **308** in up to 98% yield, and does not require the addition of either copper or Cs_2_CO_3_. In all cases, only *tert*-butyl and cyclohexyl isocyanide were successfully converted to the tetracyclic scaffolds **307** and **308**.

**Scheme 83:**
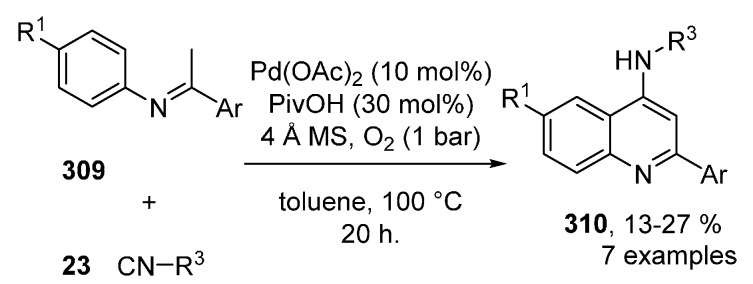
Oxidative isocyanide insertion in *N*,1-diarylethan-1-imines featuring double C-H functionalization.

In 2013, our group reported the first palladium-catalyzed imidoylative double C-H functionalization of *N*,1-diarylethan-1-imines **309**, affording the useful 4-aminoquinoline scaffold [134]. Although the yields of 4-aminoquinolines **310** are low, we were able to specifically direct imidoylative cross-coupling to two carbon nucleophiles, using molecular oxygen as a sustainable stoichiometric oxidant (Scheme 83). The yield of aminoquinazolines **310** decreases to only 13% if isopropyl isocyanide is employed (1 example).

**Scheme 84:**
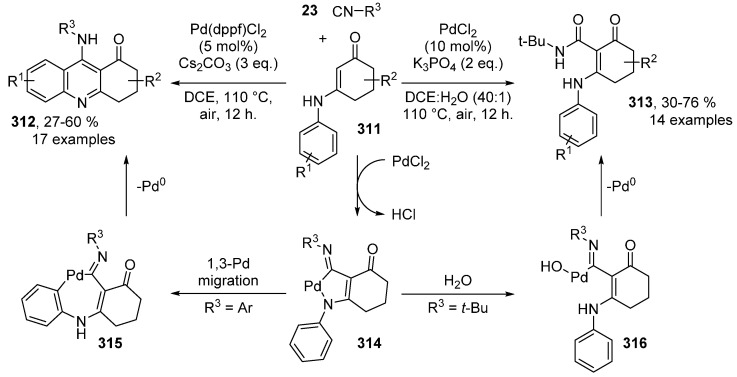
Substrate-dependent oxidative isocyanide insertion into *N*-arylenaminones.

Interestingly, a substrate-dependent chemoselective transformation with *N*-arylenaminones **311** was communicated by Luo et al. (Scheme 84) [135]. Tertiary aliphatic isocyanides afford the carboxamides **313** via hydroxyimidoylation, which was later corroborated by Luo et al. [136] However, the use of aromatic isocyanides lead to the tacrine derivatives **312** (Scheme 84). These oxidative processes proceed via the C-H functionalization of enaminone **311** and subsequent isocyanide insertion, affording the common intermediate imidoyl palladium species **314**. Bulky aliphatic isocyanide substituents induce a rapid ligand exchange between chloride and water in intermediate **314**, affording **316**. The 1,3-palladium migration was only observed in palladacycles **314** generated with aromatic isocyanides. The palladium N to C migration forms intermediate **315**, which affords tacrines **315** in up to 60% yield (Scheme 84).

### 3.4. Miscellaneous Pd^II^-Catalyzed Isocyanide Insertions

Imidoylations can also occur through metal-induced cyclization and *exo*-palladation of unsaturated substrates, generating a heteroaryl palladium intermediate. These intermediates can undergo various imidoylative cross-coupling, similar to examples mentioned above. These reactions are catalyzed by Pd^II^, and proceed in a redox-neutral manner, as such, these methods do not require an external oxidant to close the catalytic cycle.

**Scheme 85:**
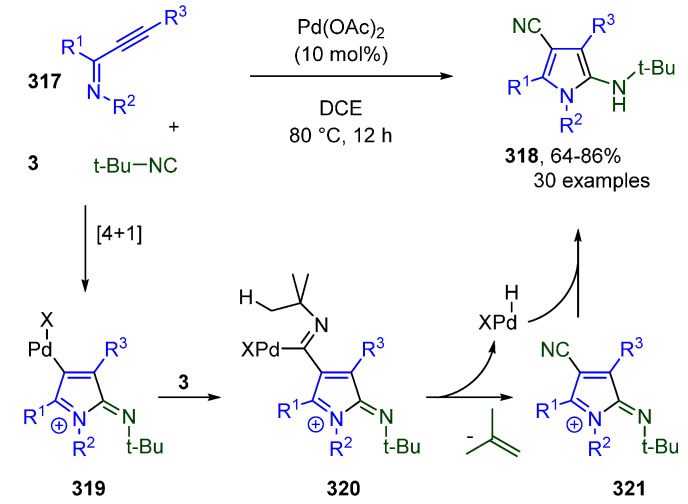
Synthesis of cyanated aminopyrroles from substituted ynimines and isoycanides. (X = OAc).

The synthesis of polysubstituted aminopyrroles proceeds via a palladium(II) catalyzed cycloimidoylation of ynimines **317** (Scheme 85) [137]. The isocyanide has a dual purpose, acting as a C_1_ building block in the initial formal [4 + 1] cycloaddition to form intermediate **319**, and as a cyanation source through de-*tert*-butylation of **320**, obtained via isocyanide insertion into the C-Pd bond of **319**. The authors propose a mechanism in which the palladium hydride expelled in the de-*tert*-butylation of **320** acts as a hydride donor (rather than undergoing reductive elimination), thus reducing the cationic intermediate **321**, thereby affording aminopyrroles **318** and regenerating Pd(OAc)_2_. While *tert*-octyl isocyanide is equally well tolerated, the reaction is incompatible with the use of primary or secondary aliphatic isocyanides. Deuteration studies indicate the aminopyrrole NH proton originates from the *tert*-butyl fragment, explaining the reason behind this incompatibility.

**Scheme 86:**
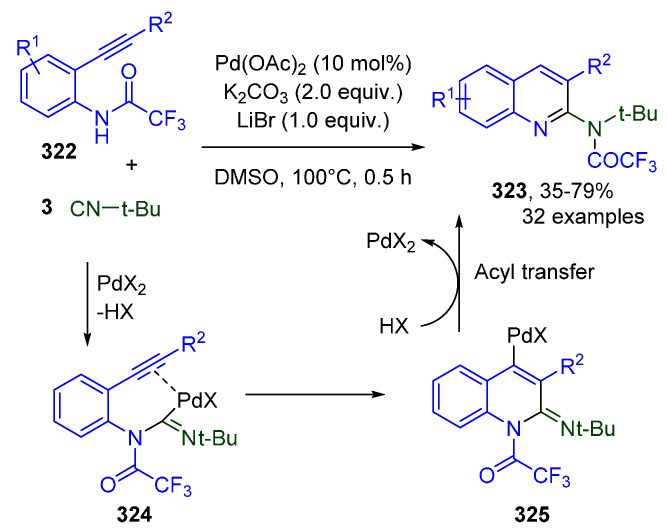
Synthesis of 2-aminoquinolines from orho-alkynylanilines and isocyanides. (X = OAc).

Another report communicated a similar synthesis of 2-aminoquinoline from *N*-trifluoroacetylated *ortho*-alkynylanilines **322** and isocyanides [138]. In this study, the authors postulated that amination of the Pd^II^ catalyst is followed by isocyanide insertion, generating intermediate imidoyl palladium species **324**, which is η^2^-bonded with the alkyne (Scheme 86). This intermediate subsequently undergoes an anti-carbopalladation presumably involving a second Pd(II) complex. The formed bicyclic carbopalladate **325** affords the 2-aminoquinolines **323** upon protodemetalation and intramolecular acyl migration (endo- to exocyclic nitrogen). This imidoylative annulation works well with tertiary isocyanides. A single secondary isocyanide was also attempted, affording the corresponding aminoquinoline in somewhat reduced yield of 40%. The same group later used this methodology to access tricyclic systems through further functionalization of the intermediate **325** [139].

**Scheme 87:**
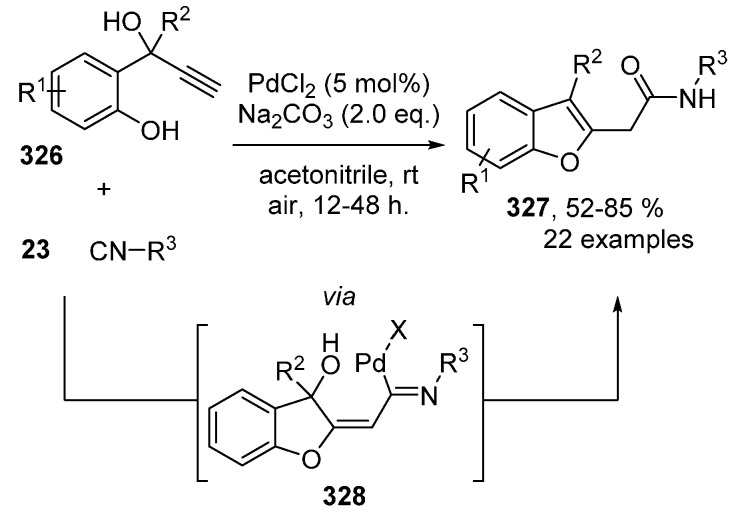
Synthesis of benzofurans via Pd-catalyzed 5-*exo*-dig cyclization and isocyanide insertion.

**Scheme 88:**
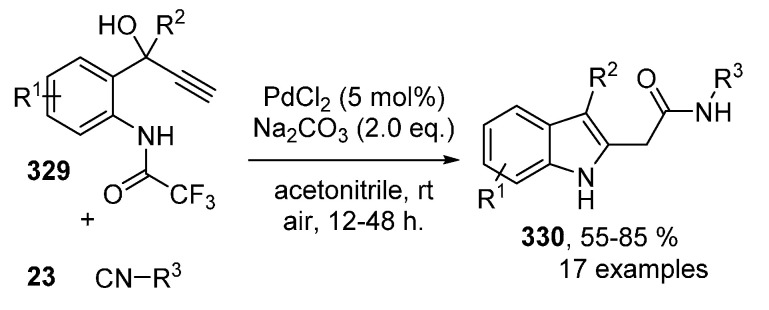
Synthesis of indoles via Pd-catalyzed 5-*exo*-dig cyclization and isocyanide insertion.

5-*Exo*-dig cyclization of 2-(1-hydroxyprop-2-yn-1-yl)phenols **326** and subsequent imidoylation affording intermediate imidoyl palladium complex **328** is proposed to lead to benzoxazoles **327** (Scheme 87). Transformation of **328** into **327** is proposed to occur via cycloalkoxylation in **328** and subsequent Pd-mediated reductive ring-opening. However, full elucidation of the reaction mechanism was not performed, and therefore, alternate mechanisms cannot completely be excluded. This transformation is equally successful with tertiary and secondary isocyanides, although the scope was not further investigated. Similarly, trifluoroacetylated aniline analogs **329** react towards indoles **330** under nearly identical conditions (Scheme 88). In this case, the trifluoroacetamide undergoes post-hydrolysis by atmospheric moisture, releasing *N*-unsubstituted indole derivatives **330**. This indole formation was only attempted with tertiary isocyanides [140].

## 4. Conclusions

Palladium-catalyzed cross-coupling reactions involving isocyanide insertions have matured significantly over the past decade. With an increasing number of intermolecular examples, it is clear that the scope of the coupling partners has also increased significantly, as new types of imidoylations catalyzed by palladium are still being reported. The main bottleneck in utilizing palladium for isocyanide insertions still appears to have unpredictable compatibility in various functionalized isocyanide reactants. Although here, too, some impressive advancements have been made in this aspect, replacing tertiary isocyanides with other isocyanides is still challenging. Additionally, imidoylative cross-couplings are increasingly compatible with other occurring cascade processes, opening new avenues for the ever-improving generation of molecular complexity in a single reaction vessel. With the progress in this field made, there have been more and more cases of imidoylative MCRs where the cross-coupling partners no longer need to be linked to achieve high selectivity and efficiency. Continued improvements on all these facets of palladium-catalyzed isocyanide insertions are necessary to increase the synthetic applications of these transformations, and to utilize imidoylative pathways in medicinally relevant synthetic procedures. It is our hope that the coming decade will see further improvements in the field of palladium-catalyzed imidoylations, leading to even more robust reactions, i.e., allowing milder reaction conditions and a broader isocyanide scope, leading to a wider coverage of chemical space.
